# Spatial Control of Microbial Pesticide Degradation in Soil: A Model-Based Scenario Analysis

**DOI:** 10.1021/acs.est.2c03397

**Published:** 2022-09-27

**Authors:** Erik Schwarz, Swamini Khurana, Arjun Chakrawal, Luciana Chavez Rodriguez, Johannes Wirsching, Thilo Streck, Stefano Manzoni, Martin Thullner, Holger Pagel

**Affiliations:** † Department of Physical Geography, Stockholm University, 10691 Stockholm, Sweden; ‡ Bolin Centre for Climate Research, Stockholm University, 10691 Stockholm, Sweden; ¶ Institute of Soil Science and Land Evaluation, Biogeophysics, University of Hohenheim, 70599 Stuttgart, Germany; § Department of Environmental Microbiology, Helmholtz Centre for Environmental Research (UFZ), 04318 Leipzig, Germany; ∥ Department of Ecology and Evolutionary Biology, University of California Irvine, Irvine, California 92697, United States; ⊥ Institute of Soil Science and Land Evaluation, Soil Biology, University of Hohenheim, 70599 Stuttgart, Germany; # Federal Institute for Geosciences and Natural Resources (BGR), 30655 Hannover, Germany

**Keywords:** small-scale heterogeneity, pesticide fate, reactive transport modeling, scale transition theory, spatial moment analysis

## Abstract

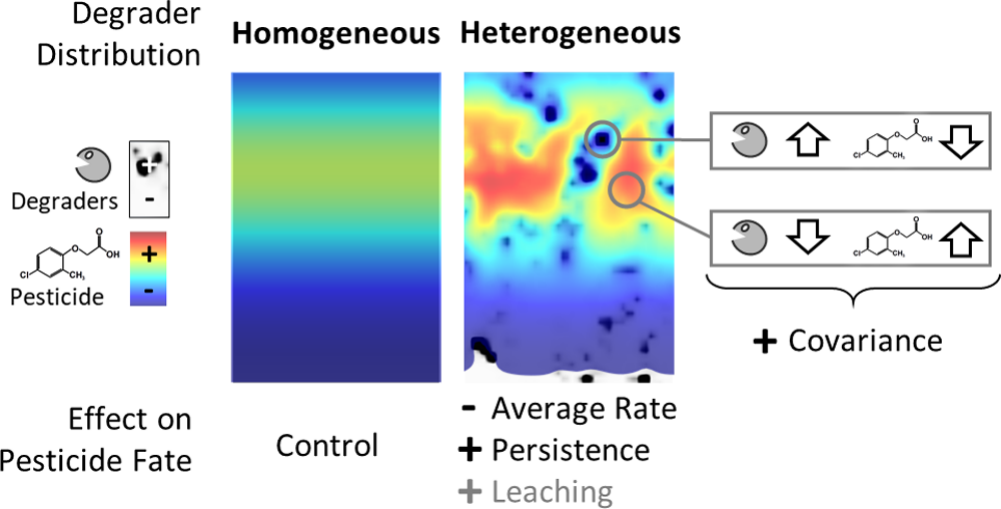

Microbial pesticide degraders are heterogeneously distributed in soil. Their spatial aggregation at the millimeter scale reduces the frequency of degrader–pesticide encounter and can introduce transport limitations to pesticide degradation. We simulated reactive pesticide transport in soil to investigate the fate of the widely used herbicide 4-chloro-2-methylphenoxyacetic acid (MCPA) in response to differently aggregated distributions of degrading microbes. Four scenarios were defined covering millimeter scale heterogeneity from homogeneous (pseudo-1D) to extremely heterogeneous degrader distributions and two precipitation scenarios with either continuous light rain or heavy rain events. Leaching from subsoils did not occur in any scenario. Within the topsoil, increasing spatial heterogeneity of microbial degraders reduced macroscopic degradation rates, increased MCPA leaching, and prolonged the persistence of residual MCPA. In heterogeneous scenarios, pesticide degradation was limited by the spatial separation of degrader and pesticide, which was quantified by the spatial covariance between MCPA and degraders. Heavy rain events temporarily lifted these transport constraints in heterogeneous scenarios and increased degradation rates. Our results indicate that the mild millimeter scale spatial heterogeneity of degraders typical for arable topsoil will have negligible consequences for the fate of MCPA, but strong clustering of degraders can delay pesticide degradation.

## Abstract 2

Strong spatial aggregation of microbial degraders at the mm-scale fosters pesticide persistence in soil.

## Introduction

Pesticides are intensively used in agriculture to sustain high yields in crop production. Their toxic nature and widespread application make them diffuse environmental pollutants that can endanger nontarget organisms and contaminate soils, air, and water.^1−4^ Residual pesticides can be found in most European agricultural soils,^5^ where they might harm beneficial soil organisms.^4,6−8^ Contamination of drinking water supplies is of special concern to human health, but despite legislative efforts pesticides are frequently found in European ground- and surface waters affecting drinking water safety.^2,9−11^ To assess pesticides’ effects on soils and water resources, their fate in soils must be quantified. Pesticides are subject to complex transport, retention, and degradation processes, making it challenging to determine whether they are dissipated, remain in the soil, or are exported into adjacent environmental compartments.^1,12−15^

Microbial degradation is the most relevant process decreasing pesticide loads in soils. Its basic prerequisite is the spatial encounter of a (bioavailable) pesticide and a competent degrader (i.e., a metabolically active microorganism able to produce a specific catalytic enzyme). In laboratory experiments spatial encounter is strongly facilitated by homogenization of soil samples. In undisturbed soils, however, pesticide degrading microorganisms are unevenly distributed and spatially aggregated at the millimeter- to centimeter-scale,^16−21^ which reduces the frequency of degrader–pesticide encounter.^22,23^ Spatial aggregation of degraders might thus impede pesticide degradation, especially considering that specific pesticide degrader microhabitats might occupy as little as 1% of the total soil volume.^23^

Small scale spatial degrader heterogeneity and its upscaled consequences for pesticide fate have so far received little attention,^18,24^ possibly because assessing the spatial variation of pesticide mineralization rates at a millimeter to centimeter scale is experimentally challenging.^18^ Only few studies have operated at these scales, mainly focusing on the phenoxy herbicides 2,4-dichlorophenoxyacetic acid (2,4-D) and 2-methyl-4-chlorophenoxyacetic acid (MCPA).^16−21,23^ For instance, Vieublé Gonod et al.,^20,21^ and Monard et al.^19^ dissected soil core slices into 6 mm × 6 mm × 6 mm cubes and found the degradation potential of 2,4-D to be aggregated in ca. 2 cm sized hotspots (which was confirmed only under wet conditions by Monard et al.^19^). The spatial discontinuity also prevails at submillimeter scales, where less than 10% of 125–500 μm sized aggregates may be colonized by 2,4-D degrading microorganisms.^23^ For MCPA, Badawi et al.^16^ found patchy, spatially variable mineralization potentials below the plow layer and identified biopores as hotspots for MCPA degradation in another study.^17^ The magnitude of spatial variation is commonly reported as coefficient of variation , where χ is a measure of mineralization potential, χ̅ is its spatial mean, and σ_χ_ is its spatial standard deviation). Observed values for small-scale variations in phenoxy herbicide mineralization potential range from *CV* = 16% to 161%.^18,20,21^

How small scale spatial degrader heterogeneities could affect pesticide fate was conceptually illustrated by Vieublé Gonod et al.,^21^ who estimated that either 2 or 75% of 2,4-D would be degraded within a 3-day incubation period, depending on whether the pesticide would be introduced to part of the soil with a low or high degradation potential. The degradation potential in soil varies along a continuum, and zones with contrasting potentials are connected by the variably saturated pore network. Driven by diffusive and convective transport, pesticides can migrate from zones of low degrader abundance to degrader hotspots. In heterogeneous soil systems, where pesticides and degraders can either be initially separated^25,26^ or pesticides become locally depleted by fast degradation at hotspots,^27,28^ diffusive transport to degrader hotspots often becomes rate limiting.^27−29^ Dechesne et al.,^27^ for instance, observed for benzoate degradation in microcosms that diffusive mass transport was slower than reaction in degrader hotspots and thus degradation rates decreased with increasing aggregation of degraders. This result brings to mind that spatially averaged reaction rates in heterogeneous systems differ from the rates calculated at the spatially averaged degrader abundance and substrate concentration.^30^ This effect is due to the nonlinear nature of the rate-concentration relation.

Chakrawal et al.^30^ and Wilson and Gerber^31^ recently applied scale transition theory to soil C cycling to describe emerging reaction rates as the sum of the mean field approximation (rates calculated from spatially averaged quantities) and additional terms considering spatial moments of substrate and degrader distributions. Both their upscaling approaches were limited to an idealized system without water flow. Lifting this restriction is crucial because advective flow can effectively alleviate transport limitation and enhance pesticide degradation.^26,29^ While typically considered as drivers of pesticide leaching,^32,33^ precipitation events could also facilitate biodegradation by increasing the contact probability between pesticide and degrading microorganisms in heterogeneous soils.^26^ Moreover, the intensity of precipitation events is important, as heavy rain can promote faster contaminant transport and reach deeper soil layers compared to low-intensity rainfall, potentially causing higher leaching than low-intensity precipitation.^32,34^

Experimental evaluation of these complex interactions and their relevance for pesticide risk assessment is challenging.^18^ This study therefore relies on reactive transport modeling as an established and powerful tool to analyze biodegradation processes and physicochemical controls.^35,36^ Using a new pesticide reactive transport model, we investigated: (i) how does spatial aggregation of microbial pesticide degraders affect pesticide dynamics? and (ii) how does the intensity of precipitation (continuous light rain vs heavy rain events) affect the degradation dynamics?

To address these questions, we simulated pesticide fate following a single application of MCPA to a soil where degraders were heterogeneously distributed to various extents. From these simulations we assessed the relevance of milliliter to centimeter scale aggregation of microbial pesticide degradation for pesticide fate at the soil column scale under unsaturated conditions. Using results from scale transition theory,^30^ we analyzed the establishment and alleviation of transport limitation under contrasting heterogeneity and precipitation scenarios.

## Materials and Methods

### Conceptual Setup

We simulated reactive pesticide transport in a soil column of 0.3 m width and 0.9 m depth (*xy*-plane) to assess the impact of spatial heterogeneity of microbial degrader distributions on pesticide degradation. Soil characteristics represented an arable Luvisol, based on a reference soil in the Ammer catchment between Tübingen and Herrenberg (Germany; 48°33′24.664″, 8°52′31.259”; see Wirsching et al.^37^ for further details). Hydraulic and degradation parameters were obtained from measured soil moisture characteristics and degradation kinetics from the same soil (Supporting Information 1 (SI1), sections 3–5). We distinguish three soil layers (0–30 cm, 30–60 cm, and 60–90 cm) by their characteristic soil hydraulic properties and refer to them as topsoil (0–30 cm) and subsoil (30–60 cm and 60–90 cm combined), respectively.

The well-studied and widely used phenoxy acid herbicide 2-methyl-4-chlorophenoxyacetic acid (MCPA) was used as a model compound. Most data on millimeter scale degradation heterogeneity is available for this pesticide and the structurally similar 2,4-dichlorophenoxyacetic acid (2,4-D).^16−21,23^ Additionally, MCPA degradation kinetics were recently analyzed in the topsoil of the reference soil.^37^ We simulated the recommended MCPA application rate (2 kg/ha). MCPA was subject to reactive transport in the soil column and only dissipated by biodegradation. Compared to native soil organic carbon (C) stocks, little C was added through the pesticide application (ca. 1.7 mg/kg C from MCPA in relation to 18.4 g/kg organic C in the top 5 cm^37^). In a previous batch experiment,^37^ growth of MCPA degraders in the reference soil was observed at an initial MCPA concentration of 20 mg/kg, but not at 5 mg/kg. In this simulation study, local MCPA concentration was much lower than 20 mg/kg. The initial MCPA concentration reached up to 9.1 mg/kg and was strongly diluted within the first day due to the applied precipitation scenarios. To keep the model parsimonious, we assumed that the MCPA degrader population was in equilibrium.

The soil was initially unsaturated and two contrasting precipitation scenarios were implemented. Seasonal variations in precipitation and evaporation were neglected, and the measured average daily net infiltration rate of 0.56 mm/d^38^ was used as a reference for precipitation scenarios.

Simulations were set up in COMSOL Multiphysics 5.5 using LiveLink for MATLAB. Values for all parameters are given in Table 1. The COMSOL  model was set up in micromolar units of C. All units were transformed to more meaningful mass-based concentrations in the Results and Discussion section by accounting for MCPA’s molar mass *n*_mol_ [g/mol] and the number of C atoms per MCPA molecule *n*_C_ [1]. Biomass concentration was expressed in gene/g, corresponding to the experimental measure, using a conversion factor *f*_*m*/*g*_ [μmol C/gene].^39^ Rates were transformed to the daily time scale.

**Table 1:** Parameter Values Used in Reactive Transport Simulations^a^

symbol	parameter	unit	values^b^	ref
**Soil Hydraulic Functions**				
θ_s_	saturated volumetric water content	1	0.49; 0.46; 0.43	measured
θ_r_	residual volumetric water content	1	0.00; 0.15; 0.00	calibrated
α_VG_	inverse of air entry value	1/m	12.30; 13.40; 3.63	calibrated
*n*_VG_	measure of pore size distribution	1	1.10; 1.12; 1.12	calibrated
*l*_VG_		1	0.5	(41)
*K*_s_	saturated conductivity	10^–5^ m/s	1.85; 24.00; 2.31	measured
**Reactive Transport**				
*D*_*m*_	MCPA molecular diffusion coefficient in water	m^2^/s	6.33 × 10^–10^	computed^42^
*K*_F_	MCPA Freundlich coefficient	μmol C/kg·(m^3^/μmol C)^*n*_F_^	1.79 × 10^–3^	(43)^c^
*n*_F_	MCPA Freundlich exponent	1	0.86	(43)^c^
λ_L_	longitudinal dispersivity	m	0.03	assumed^44^
λ_T_	transversal dispersivity	m	0.01	assumed
**Microbial Degradation**				
*B*_TS_	topsoil *tfdA* gene abundance	μmol C/kg	12.21	(37)
*f*_m/g_	conversion factor mol C/gene	μmol C/gene	1.10 × 10^–7^	(39)
γ	depth constant	1/m	3	(45)
μ_max_	maximal growth rate	1/s	2.94 × 10^–4^	calibrated
*K*_M_	monod constant	μmol C/m^3^	1.93 × 10^6^	calibrated
**Material and Chemical Properties, Global Parameters**				
ρ_F_	water density	kg/m^3^	1.00 × 10^3^	
ρ_B_	soil bulk density	10^3^ kg/m^3^	1.24; 1.32; 1.46	measured
*g*	gravitational acceleration constant	m/s^2^	9.81	
*n*_mol_	MCPA molecular weight	g/mol	200.62	
*n*_C_	C atoms per MCPA molecule	1	9	
**Geometric Variables**				
*d*_TS_	topsoil depth	m	0.3	assumed
*d*_*z*_	virtual soil column thickness	m	0.1	assumed
*d*_v,STS_	virtual soil thin section thickness	m	0.05 × 10^–3^	assumed

a Methods used to obtain measured and calibrated values are detailed in SI1.

b Where three values are given, these correspond to the 0–30 cm, 30–60 cm, and 60–90 cm soil layers, respectively. The latter values were also used for the additional 1.1 m zone.

c Measured for 2,4-D on an Orthic Luvisol.

### Governing Equations

#### Soil Hydraulic Properties and Functions

Variably saturated water flow was simulated using COMSOL’s *Richards’ Equation* module using the Mualem-van Genuchten formulation to describe soil hydraulic properties (SI1, eqs S2,S3). Soil bulk density ρ_B_ [kg/m^3^], saturated volumetric water content θ_*s*_ [1], and saturated hydraulic conductivity *K*_S_ [m/s] were measured (separately for 0–30 cm, 30–60 cm, and 60–90 cm, see SI1, section 3). The remaining van Genuchten parameters (residual volumetric water content θ_*r*_ [1], α_VG_ [1/m], and *n*_VG_ [1]) were estimated separately for each of the three layers (0–30 cm, 30–60 cm, and 60–90 cm) from fitting the van-Genuchten model of respective soil water retention curves using a hybrid (global-local) optimization algorithm (SI1, section 4). Hydraulic properties were kept uniform within each layer. Precipitation events were initiated using COMSOL’s *Events* module.

#### Reactive Transport

The advection–diffusion equation (ADE) was defined via the *Transport of Diluted Species in Porous Media* module as
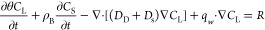
1where *R* [μmol C/m^3^/s] is the pesticide degradation rate, *C*_L_ [μmol C/m^3^] and *C*_S_ [μmol C/kg] are the dissolved phase and sorbed phase concentrations of MCPA, respectively; θ [1] is the volumetric water content and  [m/s] is the water flow velocity; *D*_D_ [m^2^/s] is the dispersion tensor and *D*_s_ [m^2^/s] is the soil diffusivity. θ [1] and *q*_*w*_ were obtained from the *Richards’ Equation* module.

The relation between *C*_L_ and *C*_S_ was described by a Freundlich sorption isotherm
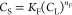
2with the Freundlich sorption coefficient *K*_F_ [μmol C/kg (μmol C/m^3^)^−*n*_F_^] and exponent *n*_F_ [1].

Soil structural heterogeneity, i.e., the spatial distribution of soil properties governing solute transport (e.g., porosity and permeability), was not considered explicitly in the simulations. Soil properties varied between the three soil depths’ layers (Table 1), but were homogeneous (uniformly distributed) within each depth layer. The macroscopic effect of smaller scale soil structural heterogeneity on solute transport was implicitly accounted for by assigning longitudinal and transversal dispersivities (λ_L_ [m] and λ_T_ [m], respectively) to *D*_D_ (SI1, section 1b). *D*_s_ accounts for reduced diffusivity in unsaturated soil using the Millington and Quirk^40^ model (SI1, eq S6).

Microbial pesticide degradation was described by the Monod-type reaction rate *R*
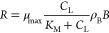
3with the maximum reaction rate coefficient μ_max_ [1/s] and the Monod constant *K*_M_ [μmol C/m^3^]. Following Chavez Rodriguez et al.,^39^ the abundance of the functional *tfdA* gene was used as a proxy for microbial MCPA degradation potential. Measured *tfdA* gene abundance in the reference soil^37^ was converted into microbial biomass *B* [μmol C/kg] using the conversion factor *f*_m/g_ [μmol C/gene] as calibrated by Chavez Rodriguez et al.^39^ Microbes were considered to be immobile, and their distributions were assigned using COMSOL’s *Domain ODEs and ADEs* module.

### Initial and Boundary Conditions

#### Water Flow

Water inflow was assigned at the upper boundary of the soil column (*y* = 0 m). The 0.9 m soil column was extended by an additional 1.1 m in the vertical *y*-direction to reduce effects from the outflow boundary. This additional zone was parametrized by the same hydraulic properties as the 60–90 cm layer. We assumed free drainage for water flow across the lower boundary (zero pressure head gradient). Initial conditions of the pressure field were obtained from spin-up simulations where the average net infiltration rate of 0.56 mm/d was set as a constant inflow boundary condition. Spin-up simulations were run until the pressure field converged to a steady state (matric potential of −0.005 MPa, corresponding to ca. 80% saturation, in the topsoil). The resulting pressure field was then applied as an initial condition in the transient simulations.

#### Pesticide Concentration

MCPA was applied at a rate of 2 kg/ha. As an initial condition this pesticide load was assigned to the upper 1.5 cm (equally distributed in horizontal direction, with a steep gradient in *y*-direction). No-flow conditions for the contaminant were assigned to all boundaries except the lower boundary where  was assigned normal to the boundary.

### Numerical Simulations

Backward differential formula time stepping with variable order of 1–5 was used to numerically solve the equations in COMSOL. A segregated solver was used for successively solving for (i) the discrete events, (ii) the pressure field, and (iii) all concentration species. The automatic Newton method was used for solving of the equations. The soil column was resolved with a 5 mm × 5 mm finite element mesh in the top 1 m. The adjacent soil column down to a depth of 2 m was resolved more coarsely with a 5 mm × 50 mm (vertical × horizontal) finite element mesh. The vertical resolution of the finite element mesh directly underneath the inflow boundary was refined by adding 15 additional horizontal element layers using COMSOL’s boundary layer function to minimize numerical errors where concentration gradients were steep. Linear elements were used for all modules. Initial conditions of MCPA, degraders, and the pressure field were loaded from gridded data files and assigned to the finite element mesh using linear interpolation. Topsoil concentrations of degrader abundances were normalized to the default value of *B*_TS_ to minimize interpolation errors.

### Microbial Distributions

A vertical profile was defined for the horizontally averaged degrader abundance  =  [μmol C/kg], where the index *i* identifies one of the *N*_*x*_ grid cells in the horizontal direction. Following Jury et al.,^45^ was assumed to be constant throughout the topsoil to a depth *d*_TS_ = 0.3m and to exponentially decrease with depth below *d*_TS_ with a factor γ [1/m]
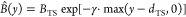
4where *B*_TS_ [μmol C/kg] is the average *tfdA* gene abundance in the topsoil of the reference soil (1.11 × 10^8^ genes/kg soil,^37^ converted with the factor *f*_m/g_ [μmol C/gene]), taken as proxy for the degrader abundance. Note that the depth *y* is 0 m at the surface and is taken positively downward.

Heterogeneous degrader distributions in the horizontal plane were created using a log-Gaussian Cox process^46,47^ (LGCP). A LGCP is a stochastic process to generate random point patterns with different degrees of clustering. Each generated point represents one degrader (one *tfdA* gene). A LGCP’s mean μ [1] is given as a function of *y* as
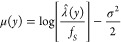
5where the *y*-dependent average intensity  [genes/m^2^] is obtained from  by accounting for the thickness of a virtual soil thin section *d*_v,STS_ [m] and *f*_S_ [genes/m^2^] is a scaling factor set to unity to obtain the nondimensional LGCP parameters. Following Raynaud and Nunan,^47^ we described the LGCP’s covariance function  using an exponential pair correlation function^46^ between two points with a separation distance *r* [m] as
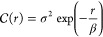
6

The LGCP can be completely defined with the intensity, variance σ^2^ [1] and scale parameter β [m].^46,47^ LGCPs were previously used to study the spatial ecology of soil microbes by Raynaud and Nunan^47^ who could successfully represent aggregated distributions of bacteria with LGCPs. Recently, Pagel et al.^48^ used spatially continuous distributions generated from LGCPs and discretized them on a 1 mm × 1 mm mesh to study the spatial control of organic C dynamics in soils. Following Pagel et al.,^48^ spatially continuous point distributions were aggregated to meet the spatial resolution of the finite element mesh used for reactive transport simulations. Spatial heterogeneity at scales smaller than the 5 mm × 5 mm mesh resolution^23,47^ was thus not taken into account. LGCPs were implemented in the R environment^49^ using the spatstat^50^ package. The parameters σ^2^ and β were manually adapted to meet published distribution metrics of phenoxy acid herbicide degradation at the millimeter scale as described in the following section.  was obtained via eq 4 and the *tfdA* gene abundance in the topsoil of the reference soil.^37^*d*_v,STS_ was adjusted to a value of 0.05 × 10^–3^ m to ensure that a realistic portion of mesh elements were colonized by MCPA degraders. Therefore, element-wise colonization ratios (CR [%]) were computed by applying a threshold of 100 genes/g soil (1/10th of the quantification limit of *tfdA* gene abundances reported in Wirsching et al.^37^) for an element to be considered as colonized. CR values were compared to percentages of microsamples colonized by phenoxy herbicide degraders observed in agricultural soils by Pallud et al.^23^

### Scenario Definition

#### Spatial Heterogeneity

Four levels of spatial heterogeneity of pesticide degraders were defined, corresponding to homogeneous (pseudo-1D) conditions (HOM), the lowest (LOW, CV = 16%),^20^ and highest (HIGH, CV = 161%)^21^ levels of spatial variability reported in literature; and an extreme case (EXTR, CV = 400%). Inclusion of the extreme scenario was motivated by the small number of studies on millimeter scale heterogeneity of microbial pesticide degraders. The available data are limited to established agricultural soils with previous or unclear exposure to pesticides (though only one study reported previous phenoxy herbicide usage).^16,19−21^ We expect that spatial heterogeneity of microbial pesticide degraders might be considerably larger in soils that were only recently converted to arable fields and received pesticides for the first time.^23^

For each scenario, 100 stochastic spatial distributions were created using characteristic σ^2^- and β-values. Their values were manually adapted to yield ensemble means of the 100 stochastic distributions close to the CV values specified for each scenario (defined for the degrader distributions *B*;  %). σ^2^ and β were further chosen in a way that the practical range (3 × the fitted range) of an exponential semivariogram fitted to the experimental semivariogram was within ±2 mm of the ≈27 mm reported by Vieublé Gonod et al.^21^ Semivariograms were produced and fitted using the gstat^51,52^ R-package (isotropic with 3.3 mm bin width and 50 mm cutoff). For each simulated scenario, fixed σ^2^- and β-values were used uniformly throughout the soil column. Individual distributions were accepted at the following limits (for topsoils, 0−30 cm)


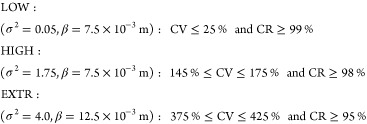


#### Precipitation

Two precipitation scenarios were defined: continuous light rain (CLR) and heavy rain events (HRE). In the CLR scenario, the measured net daily infiltration rate (0.56 mm/d^38^) was continuously applied throughout the one-year simulation period. HRE scenarios were initiated at the same infiltration rate for 2 days. After that, two day-long heavy rain events with an infiltration rate of 40 mm/d each were assigned within the first week after MCPA application (throughout day 3 and day 6). Each heavy rain event was followed by 2 days without precipitation. A continuous infiltration rate of 0.35 mm/d was applied in HRE from day 8 onward in order to achieve the same cumulative infiltration as in CLR.

### Scale Transition Theory

Upscaled effects of millimeter to centimeter scale heterogeneity of microbial degrader distributions on pesticide degradation were analyzed using the scale transition approach presented by Chakrawal et al.^30^ Their approach was used for upscaling of micrometer to millimeter scale heterogeneities in carbon cycling, but is also applicable to the millimeter to centimeter scale problem posed here for pesticide degradation. For Monod-type kinetics and biochemical homogeneity (i.e., rate parameters are constant in space), the scale transition approach provides a theoretic foundation to evaluate upscaled reaction dynamics on the basis of the spatial variance in substrate concentration  and spatial covariance between substrate and biomass concentration . With the spatial averaging operator , the second-order accurate macroscopic average reaction rate  [μmol C/m^3^/s] is given from a Taylor series expansion around  and *B̅* as
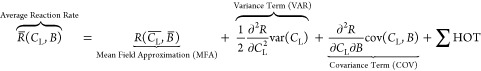
7where  [μmol C/m^3^/s] is the mean field approximation (MFA), i.e., the reaction rate in a perfectly mixed system, with *R* given by eq 3 and calculated at the spatial mean values of *C*_L_ and *B*. The partial derivatives on the right-hand side of eq 7 were likewise calculated at average quantities  (SI1, eqs S10–S12). Note that the term related to the spatial variance of *B* (i.e., ) is dropped because . We did not individually calculate the higher order terms for our analyses, but show results including their sum ∑HOT given as residual between  and the second-order accurate Taylor series expansion. The focus of our analysis was on evaluating the deviation of the observed macroscopic reaction rates between HOM and the heterogeneous scenarios that is explained by the spatial covariance term  (abbreviated COV in the following). This choice is motivated by our interest in identifying the role of microbial heterogeneous distribution, which, according to scale transition theory (for Monod-type kinetics), is captured by the covariance term.

## Results and Discussion

### Comparison of Simulated Pesticide Degrader Distributions with Experimental Observations

Stochastically generated degrader distributions showed spatial metrics (mean abundance, CV, CR) closely resembling reported values,^20,21,23,37^ especially in topsoils (SI1, Figure S3A,B,C; also see example distributions Figure S4). In the subsoil, ensemble mean CV was larger and CR lower than in the topsoil (SI1, Figure S3 D,E). While distributions were not intentionally parametrized for this behavior, it is in line with experimental observations of increased heterogeneity and lower colonization ratios in subsoils.^16^

### Influence of Hydrodynamic Dispersion on MCPA Biodegradation

Besides pesticide degrader heterogeneity, soil structural heterogeneity might be important for pesticide fate.^53^ In our simulations, we did not explicitly consider this type of heterogeneity at scales resolved by our spatial discretization (i.e., soil structural and hydraulic parameters were uniformly applied within each depth layer, Table 1). Instead, we assigned dispersivities (λ_L_ and λ_L_) to the dispersion tensor (SI1, eq S5) to capture the effective behavior of solute transport emerging from the variance in local flow velocities caused by smaller scale soil structural heterogeneities. In our simulations λ_L_ and λ_T_ were kept constant throughout the entire simulation domain and we tested how the choice of these effective parameters affected simulation outcomes. Within the tested range of 0.01 to 0.1 m for λ_L_ and for ratios of λ_L_/λ_T_ of 3 and 10, the choice of dispersivity values had little effect on MCPA biodegradation (quantified by comparing times needed for 50% removal of pesticide (DT50) from soil, see SI1, section 8), and the spread between individual realizations of the distributions at default dispersivity values was much larger. In all simulations, pesticide transport was moreover facilitated by a continuously high water saturation (ca. 80% in CLR and up to 98% in HRE during rain events).

### Implications of Spatial Degrader Heterogeneity for Pesticide Fate

**Figure 1:**
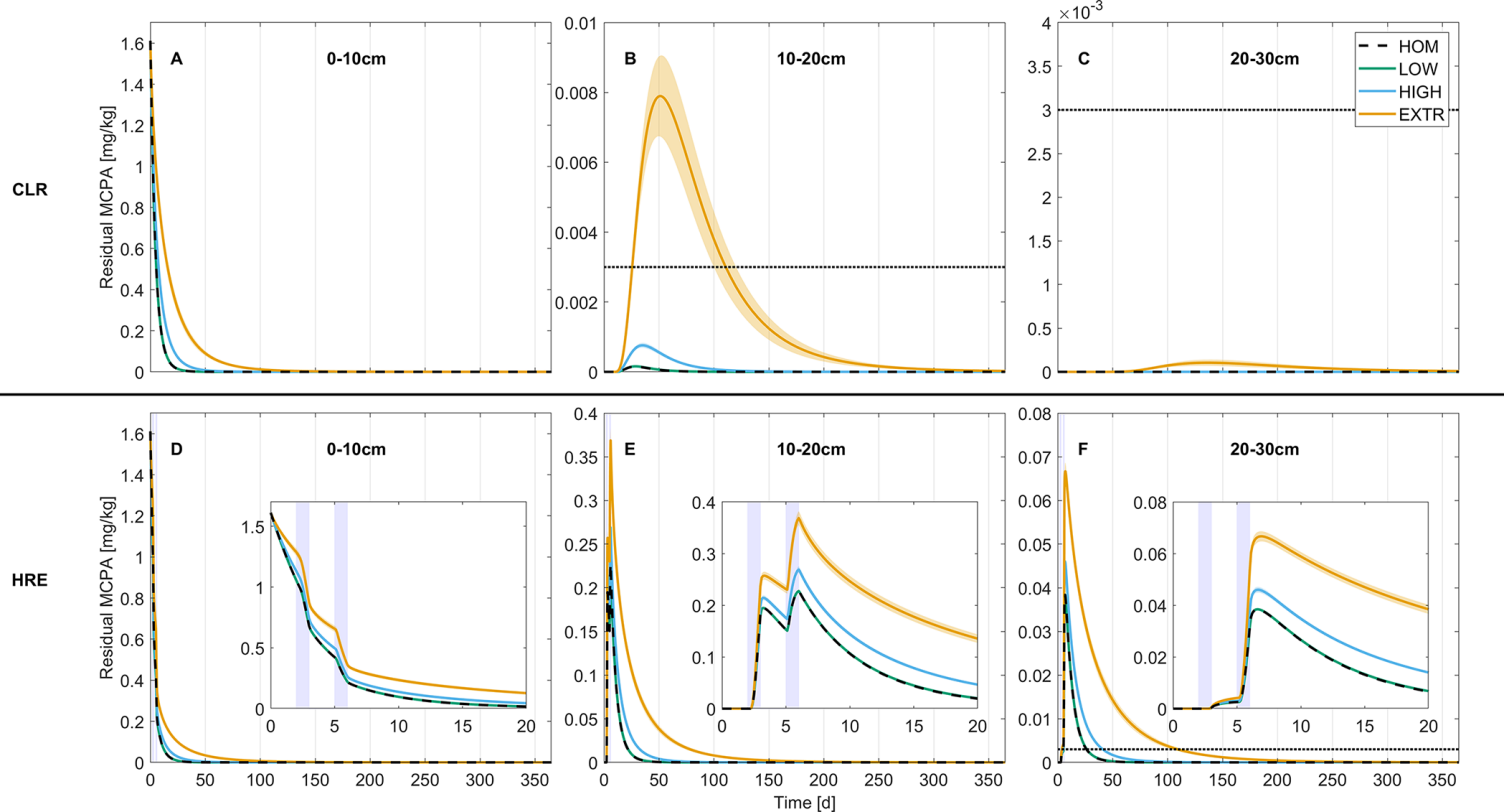
Time series of averaged residual MCPA concentrations in 10 cm depth intervals for continuous light rain (CLR; A–C) and heavy rain events (HRE; D–F) scenarios. Lines represent the scenario means, and shaded areas mark their 99%-confidence intervals. Blue bars in panels D–F indicate when heavy rain events occurred. Dotted black lines indicate a detection threshold of 3 μg/kg. Insets in panels D–F show details of days 0 to 20. MCPA concentrations below 30 cm were negligible (<3 μg/kg) and are not shown. Note that *y*-axis scales vary from panel to panel.

To elucidate the role of millimeter scale spatial degrader heterogeneity on pesticide fate we evaluated residual MCPA concentrations along the soil profile (Figure 1). Scenario outcomes were clearly distinct between the two precipitation scenarios. In the CLR scenarios, MCPA concentrations below 20 cm never exceeded the detection threshold (3 μg/kg; set to the lower end of detection limits of acidic herbicides in soil;^54^Figure 1B,C). After heavy rain events MCPA leached deeper and was detectable down to depths of 30 cm (Figure 1F) but did not reach the subsoil (data not shown).

Scenarios with low spatial heterogeneity of pesticide degraders (Figure 1, green lines) were hardly distinguishable from homogeneous simulations (dashed black lines). In turn, high and extreme heterogeneity (blue and orange lines, respectively) affected residual pesticide concentrations in both precipitation scenarios and all soil depths. Degradation in HIGH and EXTR generally proceeded slower than in HOM and LOW, resulting in higher residual MCPA concentrations at any given time. In HRE scenarios, this consistently led to MCPA being detectable for longer periods (21, 21 ± 0, 36 ± 0, and 103 ± 2 days [mean ± standard error of the mean] at a depth of 20−30 cm for HOM, LOW, HIGH, and EXTR, respectively). In CLR scenarios, only in extremely heterogeneous scenarios (EXTR) was MCPA detectable below the uppermost 10 cm and remained detectable for 73 ± 4 days at a depth of 10–20 cm.

**Figure 2:**
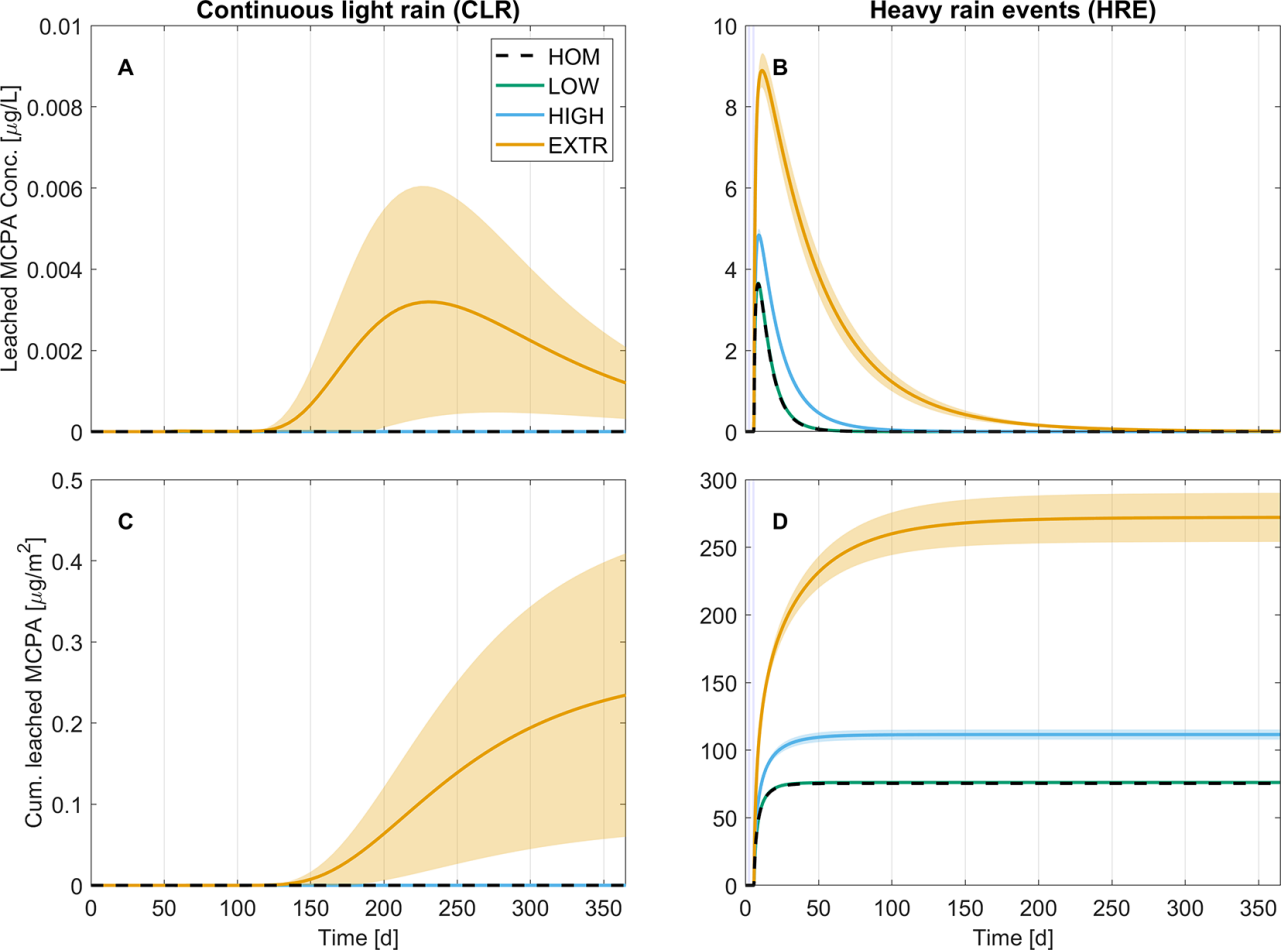
Time series of averaged MCPA leachate concentration (A,B) and cumulatively leached MCPA load (C,D) from the topsoil at 30 cm depth in continuous light rain (CLR; A,C) and heavy rain events (HRE; B,D) scenarios. Lines represent scenario means and shaded areas mark their 99%-confidence intervals. Blue bars in panels B and D indicate when heavy rain events occurred. Note that *y*-axis scales vary from panel to panel.

MCPA leaching within the soil column was minimal and only after heavy rain events notable amounts of MCPA (concentration in the μg/L range) were leached from the topsoil (Figure 2). At most, MCPA leaching amounted to ≈0.1% of the initial application rate of 2 kg/ha (Figure 2D). In the subsoil MCPA leaching was all together untraceable (data not shown).

The spatial heterogeneity of degraders had less impact on MCPA dynamics than precipitation, but leaching of MCPA and leachate concentrations consistently increased with degrader heterogeneity within a given precipitation scenario. This is in line with simulation results of microbial nutrient cycling in spatially heterogeneous domains under fully saturated conditions.^55^ In our study, with extreme spatial heterogeneity of degraders, leaching from the topsoil was still ongoing at the end of the one year simulation period, however, only at low ng/L leachate concentrations (Figure 2A,B and SI1, Figure S6). Despite these low concentrations, MCPA persistence until the next cultivation cycle might pose the risk of accumulation in the subsoil following several MCPA applications. However, degradation of phenoxy herbicides in soils generally occurs more rapidly after repeated applications due to the enrichment and dispersal of a specific degrader population.^23,56,57^ Neither of these processes was considered in our model, so that no general conclusion regarding MCPA fate after repeated applications can be drawn from this study.

A direct comparison of our simulation results to field studies is difficult because of the lack of experimental data measuring the impact of millimeter scale degrader heterogeneity on pesticide fate at the soil column scale. With experimental data we could test if the simplifications made in our model (e.g., preferential flow and surface runoff were neglected) affect model performance. Compared to a previous modeling study by Rosenbom et al.,^24^ our simulations predicted slightly higher MCPA leaching. While in our simulations even in CLR we observed leachate concentrations of up to 3 ng/L from 30 cm, Rosenbom et al.^24^ observed leaching of MCPA (with concentrations ≥ 1 ng/L) by matrix flow only if biodegradation was neglected, and only at a depth of 24 cm and no longer at 31 cm. In their simulations, soil was initially less saturated and precipitation events were taken from field observations, where no considerable rain events occurred within 2 weeks after the simulated MCPA application. Both aspects likely resulted in MCPA being more mobile in our scenario simulations, which promoted MCPA leaching.

Despite this discrepancy in precipitation scenarios, results of heterogeneous degradation scenarios are comparable between the two studies. Rosenbom et al.^24^ evaluated degradation scenarios with spatially randomly distributed MCPA degradation potentials (note that this is not the same as the aggregated distributions used for simulations here). They derived heterogeneous degrader distributions from the experimental data of Badawi et al.,^16^ who reported a homogeneously distributed degradation potential in the topsoil (8 cm depth) and an increased spatial heterogeneity with a CV above 50% in the transition zone (28 cm depth).^16^ The heterogeneous scenario of Rosenbom et al.^24^ is thus closest to our LOW scenario. In line with their results, we found leachate concentrations from 30 cm in LOW scenarios with CLR to never exceed 1 ng/L (Figure 2A).

The combined evidence of these previous results and our simulations exploring the full range of observed and expected spatial heterogeneity suggests that spatial aggregation of degraders is negligible in assessing MCPA leaching from the subsurface. However, our results indicate that the patchy distribution of degraders can modulate MCPA leaching within the topsoil and substantially influence how long MCPA remains detectable in soils and consequently how long soil organisms might be exposed to the chemical. Though the actual risk of exposure is also influenced for instance by abiotic inactivation mechanisms such as sorption.

Little is known about the spatial distribution of degraders of other pesticides. MCPA typically sorbs weakly and degrades fast in soils. How spatial degrader heterogeneity affects the reactive transport of more strongly sorbing compounds with slower biodegradation half-lives needs further assessment. In the absence of experimental evidence, our framework can be used to explore expected and worst case scenarios for other pesticides by altering the physicochemical parameters and adapting application rates and expected degrees of aggregation (i.e., by tuning the parameters of the LGCP).

### Spatial Heterogeneity Controls of Macroscopic Reaction Rates

All else being equal, the degradation rates in increasingly more heterogeneous scenarios decrease due to transport limitation at small scale arising from the aggregation of degraders.^27^ Transport limitation establishes if the transport of a substrate to a location of high degradation potential proceeds slower than its degradation at this location. In more heterogeneous scenarios, degraders are concentrated in fewer ’hotspots’ leaving larger areas of the soil void of degradation potential (statistically captured in the CV-value). On average, the substrate thus needs to be transported over a longer distance to arrive at a degrader hotspot. Transport to such hotspots thus takes longer while degradation at hotspots proceeds faster than in homogeneous scenarios. The extent of transport limitation is eventually determined by the specific parameter values assigned to transport and reaction processes, but modulated by this characteristic transport distance.

With spatial heterogeneity, substrate concentrations rapidly deplete in degradation hotspots, but remain higher in spots with low degradation potential.^15^ Consequently, reaction rates computed from average biomass and substrate concentrations–the mean field approximation (MFA)^30^ – overestimate the macroscopic (i.e., spatially averaged) reaction rates in these heterogeneous systems. A similar argument has also been derived from experimental observations of atrazine degradation.^28^ Recently, Chakrawal et al.^30^ linked decreased reaction rates in heterogeneous systems to spatial moments of degrader and substrate distributions by a dynamic upscaling approach derived from scale transition theory (eq 7). Expressed with this framework, the preferential depletion of substrate at locations of high degrader abundance manifests in a spatial anticorrelation between biomass and substrate distributions. This attribute is captured by a negative covariance term (COV) in eq 7, explaining a slowing down of the overall reaction rate.

It is important to recall that our heterogeneity scenarios differ only with respect to the microbial distribution. Our ’homogeneous’ HOM scenario does not represent a well-mixed system as the MFA, because also in HOM MCPA varies vertically; that is, the spatial variance of the substrate distribution is nonzero. This effect is captured in the variance term (VAR) of eq 7 (which also has a negative sign; see SI1, Figure S7). In HOM, COV = 0 as there is no variance in the spatial distribution of degraders. According to scale transition theory (eq 7), the deviation of *R̅*–MFA from 0 is then explained by the spatial variance in substrate distribution (VAR) and higher order terms (*∑*HOT). The latter can be calculated as *∑*HOT = *R̅*–MFA – COV – VAR.

**Figure 3:**
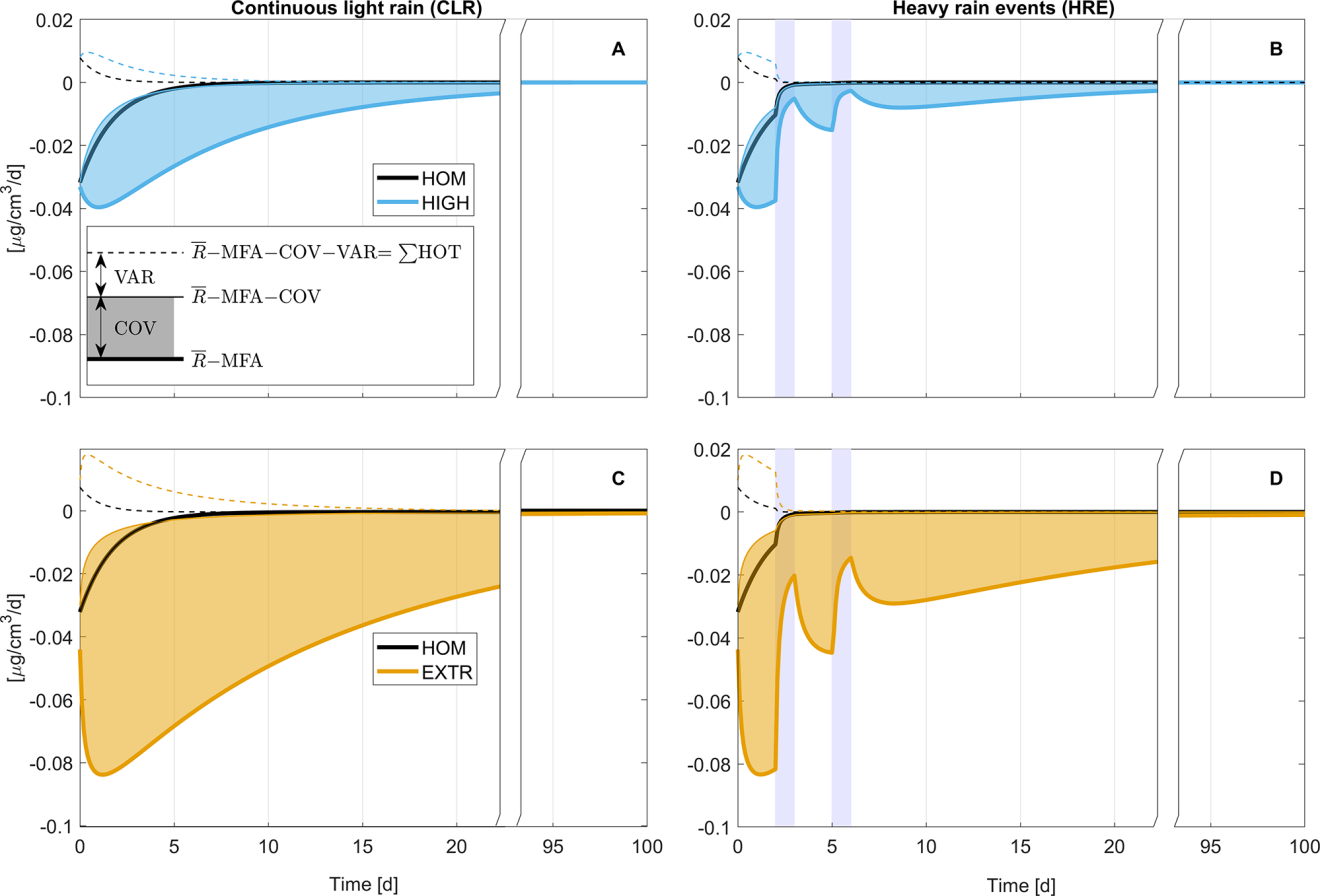
Temporal evolution of the difference between *R̅* and MFA (*R̅*–MFA) in HOM, HIGH (A,B), and EXTR (C,D) in continuous light rain (CLR; A,C) and heavy rain events (HRE; B,D) scenarios. The contribution of COV is represented by shaded areas, and the difference between their top edge and the dashed lines represents the contribution of VAR (compare inset in panel A). The deviation of the dashed line from 0 is explained by higher order terms (*∑*HOT, the remainder of eq 7). All measures represent ensemble means computed from simulation outputs. Temporal dynamics and 99%-confidence intervals of *R̅*, COV, and VAR are presented in SI1, Figure S7. Blue bars indicate when precipitation events occurred. Note that the *x*-axes is discontinued between day 20 and 95.

Figure 3 visualizes the contributions of the different terms in eq 7. Results for LOW are not shown because they were indistinguishable from HOM (compare the temporal evolution of individual terms *R̅*, COV, and VAR in SI1, Figure S7). Thick lines in Figure 3 represent, for each scenario, the difference between the macroscopic reaction rate *R̅* in the topsoil and the reaction rate predicted from averaged quantities, the MFA. In line with theory and the previous observations, *R̅*–MFA was more negative in more heterogeneous scenarios (in order HOM < HIGH < EXTR, respectively, shown as black, blue, and orange thick lines in Figure 3; also see SI1, Figure S8). This result confirms that macroscopic rates were considerably lower in more heterogeneous conditions (see also *R̅* in SI1, Figure S7). However, given the transient nature of our simulations, it is not possible to derive a unique relationship describing the deviation from the MFA as a function of the spatial degrader variability CV alone (SI1 section 11 and SI1, Figure S8B). It is important to note that the eventual convergence to *R̅*–MFA = 0 is a result of the decline in absolute values of rates (as MCPA is used up), while the effect of heterogeneity remains up to the end of the simulations, as illustrated by the dimensionless scale transition correction ((*R̅*–MFA)/MFA) (SI1, Figure S8) suggested by Wilson and Gerber.^31^

In line with our prediction from scale transition theory, differences between HOM and heterogeneous scenarios are largely attributed to the covariance terms (shaded areas in Figure 3). However, in the initial phase of the simulations also VAR (areas between thin solid and thin dashed lines in Figure 3) and *∑*HOT (thin dashed lines in Figure 3) effects are important in all scenarios. As time progresses, VAR and *∑*HOT effects decrease faster than COV effects. Markedly, in HRE scenarios, heavy rain events promote further substrate dispersion, resulting in even faster reductions of VAR as MCPA is evenly distributed in the topsoil. Similarly, *∑*HOT are reduced because they as well depend on the local deviation of MCPA concentrations from its macroscopic mean . Moreover, as MCPA is degraded *C*_L_ becomes much smaller than *K*_M_ leading to approximately multiplicative degradation kinetics (i.e., *C*_L_/(*K*_M_ + *C*_L_) ≈ *C*_L_/*K*_M_ in eq 3). As also shown by Chakrawal et al.,^30^ for multiplicative kinetics VAR and *∑*HOT terms vanish and all deviation from the MFA is captured by the covariance term alone. In all scenarios, the absolute values of the combined COV and VAR terms are thereby at least five times as large as *∑*HOT. These results demonstrate that COV well explains deviations between reaction rates in HOM and heterogeneous scenarios, but also that considering COV and VAR together can reduce the error of estimating heterogeneous reaction rates from the MFA.

Dynamics in HRE provide an interesting test case displaying the relations between degradation, transport, and the emerging covariance dynamics. Like in CLR, previous to heavy rain events, *R̅*–MFA and COV in HIGH and EXTR become drastically more negative as MCPA is preferentially depleted at locations with high degrader density as diffusive transport fails to supply sufficient amounts of MCPA from locations with lower degrader density. In contrast, during heavy rain events MCPA becomes rapidly redistributed, supplying degrader hotspots again with higher substrate concentration (example simulations in SI2, Figures S9–S12). This homogenization manifests in a reduced magnitude of COV and consequently *R̅*–MFA. The same cycle occurs once again as the first heavy rain event ceases, and advective and dispersive transport of MCPA stall.

The consistency with which the covariance term COV explains decreased reaction rates in systems where degraders are distributed heterogeneously (i.e., soils) compared to such systems where degraders are distributed homogeneously (i.e., batch experiments) suggests that COV is an indirect metric of transport limitation. Thus, COV can be used to more accurately transfer degradation rates measured in homogenized soil samples to naturally heterogeneous soils in the field. Even though Chakrawal et al.^30^’s approach was originally developed for systems with only negligible transport, we display in this work that it also holds under reactive transport conditions. However, empirical data on the magnitude of COV is limited, and its determination outside of such idealized simulation studies appears largely impractical. While studies carried out in fully saturated conditions have indicated that using travel time of solutes may close the gap on estimating bulk reaction rates,^55,58,59^ the estimation of the same in unsaturated settings remains elusive.

### Pesticide Remobilization after Heavy Rain Events

**Figure 4:**
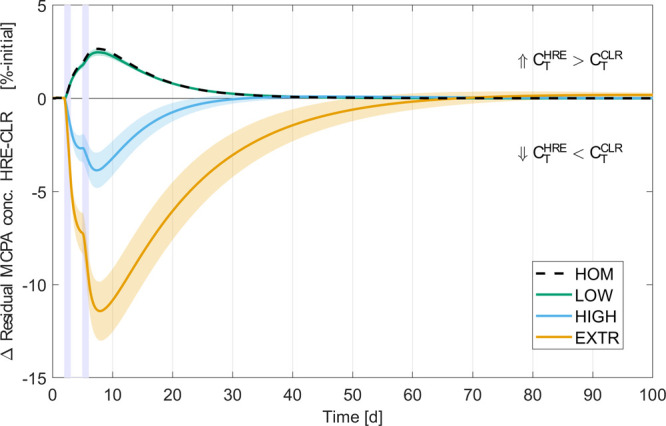
Time series of the difference in averaged residual MCPA concentration in the entire simulation domain between heavy rain events (HRE) and continuous light rain (CLR) scenarios. The difference is computed as  and normalized by the initial MCPA concentration *C*_*T*_(*t* = 0). Positive and negative values indicate whether more MCPA remained in HRE or CLR scenarios, respectively. Lines indicate heterogeneity scenario means and shaded areas around them are 99%-confidence intervals. Blue bars mark times during which heavy rain events in HRE scenarios occurred.

Two counteracting effects of heavy rain events were observed in this study. On the one hand, in heterogeneous scenarios heavy rain events caused (minor) pesticide leaching to the subsoil where degrader concentrations were lower. On the other hand, heavy rain events temporarily alleviated transport limitations, thereby increasing degradation rates in the topsoil in heterogeneous (HIGH and EXTR) scenarios. To evaluate the net effect of heavy rain events on pesticide fate in homogeneous and heterogeneous scenarios, MCPA concentration time series in the entire domain were analyzed (Figure 4).

A strongly diverging behavior was observed between HOM/LOW and HIGH/EXTR. If degraders were distributed more homogeneously, heavy rain events decreased pesticide degradation compared to CLR and residual concentrations were temporarily increased . Rather than MCPA leaching, which was negligible, the more homogeneous distribution of MCPA in the topsoil was largely responsible for the slower degradation in HRE compared to CLR scenarios. This effect is caused by two processes. First, dilution due to higher saturation after rain lowers MCPA concentrations and thus reaction rates. Second, the applied Freundlich sorption isotherm (eq 2) describes the relationship between sorbed (*C*_S_) and the dissolved (*C*_L_) MCPA with a power law as  with the exponent 0 < *n*_F_ ≤ 1. Consequently, as *C*_L_ decreases, proportionally more substrate is sorbed than at higher values of *C*_L_. By distributing MCPA more equally within the soil column, more MCPA becomes sorbed and inaccessible to microbial degradation, thereby reducing degradation rates in HRE scenarios.

These two processes were also active in the heterogeneous scenarios; however, in HIGH and EXTR initially large negative values  were observed. In these scenarios, heavy rain events strongly facilitated degradation, which is especially effective in the EXTR scenario. Even after rain events had ceased for 26 and 64 days in HIGH and EXTR, concentrations remained lower than in CLR despite some pesticide having leached to the subsoil. This sustained effect might not only be due to the remobilization and mixing of pesticide, but also to the rapidly increasing water content in the soil column. While the soil was initially moist (83% saturation), during heavy rain events almost full saturation was reached (98%), facilitating solute diffusion (SI1, eq S6^40^). With the applied Millington and Quirk^40^ tortuosity model (SI1, section 1b) and the defined parameter values, the increase from 83 to 98% saturation increased the soil solute diffusion coefficient by more than 70%. By removing transport limitations in the heterogeneous scenarios, this moisture increase thus promoted degradation. These results are in line with experimental studies, as for example, Dechesne et al.^27^ observed a drastic reduction of benzoate degradation rates with decreasing water content in artificial systems with heterogeneously distributed degraders and Monard et al.^19^ observed a similar effect for 2,4-D degradation in soil.

### Limitations and Implications

The macroscopic effects of small scale spatial degrader heterogeneity on pesticide degradation in soil has received little attention so far,^18,24^ and potential effect ranges have not been explored before. With this study we contribute to closing this knowledge gap. In line with a previous study,^24^ we found that degrader spatial heterogeneity has negligible effects on MCPA leaching from subsoil; however, heterogeneous degrader distributions can slow down MCPA degradation within the topsoil, leading to increased persistence. Our analysis was limited to a few heterogeneity scenarios and a single pesticide (MCPA). The abundance of the different levels of spatial degrader aggregation in natural soils remains elusive, and even the absolute ranges of heterogeneity measures (e.g., CV and semivariogram ranges) have hardly been explored.^18^ Such investigations would be particularly important across soil management gradients, encompassing, for example, no-till systems and extensively managed soils, as well as for pesticides with physicochemical properties different from those of MCPA. This might be achieved by extending existing sampling methods (e.g., Badawi et al.^16^). Predicting the impact of spatially variable degradation potentials combined with different substrate transport regimes remains a challenge. We approached it by analyzing spatial moments of substrate and degrader distributions and found the covariance term derived from scale transition theory to be an indirect metric of transport limitation and a quantitative predictor of reduced degradation rates in heterogeneous systems. Our analysis was limited to Monod-type degradation kinetics that converged to multiplicative degradation kinetics as substrate was consumed. Modeling contaminant degradation with more general kinetic equations, such as the equilibrium chemistry approximation,^60^ could yield additional insights on heterogeneity effects when contaminant and biomass concentrations vary strongly through time. Further developing these approaches will provide a way forward to disentangle multiple nonlinear processes across shifting system states. Linking the scale transition approach of reaction kinetics with upscaling approaches for reactive transport could lead to further progress in evaluating the implications of small-scale variability of biodegradation in heterogeneous soils on pesticide fate and management at the field scale.

## Supporting Information Available

Additional details on reactive transport equations; equations describing the individual terms of the scale transition; methods used to obtain measured and calibrated parameter values; properties and examples of the generated stochastic distributions; model exploration with varying dispersivity values; time series of individual scale transition terms; dimensionless representation of scale transition approach (PDF)
Example simulations (pptx)

## Notes

Codes used to generate heterogeneous distributions of microbial degraders and to setup the reactive transport framework in COMSOL Multiphysics   using LiveLink   for MATLAB   are freely available at 10.5281/zenodo.6532270.
