# Supplementary material for: Spatial Control of Microbial Pesticide Degradation in Soil: A Model-Based Scenario Analysis
Source: Environ Sci Technol. 2022 Sep 27;56(20):14427–38. doi: 10.1021/acs.est.2c03397 (PMC9583605; doi:10.1021/acs.est.2c03397)
Supplement: Supplementary file 1 — es2c03397_si_001.pdf [file es2c03397_si_001.pdf]

Supporting Information 1 for

Spatial Control of Microbial Pesticide Degradation in Soil: A model-based Scenario Analysis

Erik Schwarz*[,1,2,3], Swamini Khurana[1,4], Arjun Chakrawal[1,2], Luciana Chavez Rodriguez[3,5], Johannes Wirsching[6], Thilo Streck[3], Stefano Manzoni[1,2], Martin Thullner[4,7] and Holger Pagel[3]

[1]Department of Physical Geography, Stockholm University, 10691 Stockholm, Sweden

[2]Bolin Centre for Climate Research, Stockholm University, 10691 Stockholm, Sweden

[3]Institute for Soil Science and Land Evaluation, Biogeophysics, University of Hohenheim, 70599 Stuttgart, Germany

[4]Department of Environmental Microbiology, Helmholtz Centre for Environmental Research (UFZ), 04318 Leipzig, Germany

[5]Department of Ecology and Evolutionary Biology, University of California Irvine, Irvine, California 92697, United States

[6]Institute for Soil Science and Land Evaluation, Soil Biology, University of Hohenheim, 70599 Stuttgart, Germany

[7]Federal Institute for Geosciences and Natural Resources (BGR), 30655 Hannover, Germany

*Corresponding author: erik.schwarz@natgeo.su.se

15 Pages

0 Tables

8 Figures

Table of Contents

1. Governing Equations S3
a. Water transport S3
b. Reactive Transport S3
2. Scale transition theory S4
3. Measurements of soil water retention curves, hydraulic conductivities and bulk densities S5
4. Estimation of soil hydraulic parameters S5
5. Fitting of the MCPA degradation model S6
6. Distribution properties S8
7. Distribution examples S9
8. Model exploration for different dispersivity values and ratios S10
9. Leachate concentration and cumulatively leached MCPA load S11
10. Temporal evolution of scale transition terms S12
11. Dimensionless scale transition approach S13
12. References S15

List of Figures

Figure S1 Fitted SWRC against measured data S6
Figure S2 Fits of degradation kinetics to experimental data S7
Figure S3 Distribution statistics of created distributions S8
Figure S4 Exemplary distributions of degrader abundances of heterogeneity scenarios S9
Figure S5 Sensitivity of DT50 values to variation in longitudinal and transversal dispersivities S10
Figure S6 Time series of MCPA leachate concentration and cumulatively leached MCPA load S11
Figure S7 Temporal evolution of terms in the scale transition S12
Figure S8 Dimensionless scale transition correction S14

1. Governing Equations

a. Water transport

Water flow was simulated using the *Richards' Equation* module in COMSOL Multiphysics® 5.5 with the Richards' equation for incompressible fluids and zero storage in the absence of any sinks and sources given in the head-based form as

$$C_m \frac{\partial h}{\partial t} - \nabla \underbrace{K(\Theta) \cdot \left(\nabla h + \begin{pmatrix} 0 \\ 1 \end{pmatrix} \right)}_{=q_w} = 0\,, \tag{S1}$$

with the specific moisture capacity C_m [1/m], the pressure head h [m] and water flow velocity q_w [m/s], where the unsaturated hydraulic conductivity $K(\Theta)$ [m/s] is described by the Mualem-van Genuchten model

$$K(\Theta) = K_s \Theta^{l_{VG}} \left(1 - \left(1 - \Theta^{\frac{1}{m}}\right)^m\right)^2, \tag{S2}$$

as a function of the effective saturation Θ [1]

$$\Theta = \frac{\theta - \theta_r}{\theta_s - \theta_r} = \frac{1}{(1 + (\alpha_{VG} h)^{n_{VG}})^m}, \tag{S3}$$

with $l_{VG} = 0.5$ [1] and $m = 1 - \frac{1}{n}$ [1] (Mualem, 1976).

The residual volumetric water content θ_r[1] as well as the van Genuchten parameters α_{VG} [1/m] and n_{VG} [1] where obtained from fitting measured soil water retention curves (SWRC) (section 4). Values of saturated hydraulic conductivities K_S [m/s] and saturated volumetric water contents θ_s [1] where directly measured (section 3).

b. Reactive Transport

The advection-diffusion-reaction equation (ADR) was defined using COMSOL®'s *Transport of Diluted Species in Porous Media* module as given by Eq. 1 in the main text

$$\frac{\partial \theta C_L}{\partial t} + \rho_B \frac{\partial C_S}{\partial t} - \nabla \cdot [(D_D + D_S)\nabla C_L] + q_w \cdot \nabla C_L = R\,, \tag{S4}$$

where ρ_B [kg/m^3] is the soil bulk density and the relation between solution phase pesticide concentration C_L [μmol C/m^3] and the sorbed phase concentration C_S [μmol C/kg] was defined with the Freundlich sorption isotherm given by Eq. 2 in the main text as $\mathrm{C_S} = \mathrm{K_F}(\mathrm{C_L})^{\mathrm{n_F}}$, with the Freundlich coefficient K_F $[(\mu\mathrm{mol\ C/kg})(\mu\mathrm{molC/m^3})^{-n_F}]$ and exponent n_F [1]. Spatial heterogeneity in the hydraulic parameters was not explicitly considered in our modeling framework and parameters were kept uniform in each depth layer. The dispersion tensor $D_D = \begin{pmatrix} D_D^{xx} & D_D^{xy} \\ D_D^{yx} & D_D^{yy} \end{pmatrix}$ [m^2/s] captures the effective behavior of solute transport that emerges from the variance in local flow velocities caused by smaller scale structural heterogeneities. D_D was defined in COMSOL® as

$$\begin{aligned} D_D^{xx} = D_D^{yy} &= \lambda_L \frac{q_{w,x}^2}{|q_w|} + \lambda_T \frac{q_{w,y}^2}{|q_w|}, \\ D_D^{xy} = D_D^{yx} &= (\lambda_L - \lambda_T)\frac{q_{w,x} q_{w,y}}{|q_w|}\,, \end{aligned} \tag{S5}$$

where λ_L [m] and λ_T [m] are the longitudinal and transversal dispersivity, respectively, and $q_w = \begin{pmatrix} q_{w,x} \\ q_{w,y} \end{pmatrix}$ [m/s] is the velocity vector. No measurements for λ_L and λ_T where available and a value of

$\lambda_L = 0.03$ m was assumed (Vanderborght and Vereecken, 2007). To test the sensitivity of the model to the choice of λ_L, its value was varied to 0.01, 0.05 and 0.1 m and times needed for 50% degradation in the different heterogeneity scenarios were compared (supplementary Figure S4). We further assumed a ratio of $\frac{\lambda_L}{\lambda_T} = 3$ but also tested ratios of $\frac{\lambda_L}{\lambda_T} = 10$. A uniform value for both λ_L and λ_T was assigned to the entire simulation domain.

The soil diffusion coefficient D_S [m^2/s] accounts for decreased diffusivity under unsaturated conditions. This was accounted for in COMSOL® using the Millington and Quirk (1961) model

$$D_s = \frac{\theta^{10/3}}{\phi^2} D_m \,, \tag{S6}$$

with the molecular diffusion coefficient of MCPA D_m [m^2/s] and the porosity $\phi \coloneqq \theta_s$.

The pesticide degradation rate R [μmol C/m^3/s] was given by Eq. 3 in the main text and was a function of microbial degrader concentration B [μmol C/kg]. Microbes were considered to be in steady-state and non-mobile $\left(\frac{dB}{dt} = 0\right)$. Degrader distributions were assigned via the *Domain ODEs and ADEs* module in COMSOL®. To allow for mass-balance closure a "degraded-C" pool (CO_2 [μmol/kg]; i.e., assuming 100% of the degraded MCPA evolves as CO_2) was considered as

$$\frac{\partial CO_2}{\partial t} = \frac{1}{\rho_B} R \,. \tag{S7}$$

Mass balance in the entire soil column domain Ω (with the virtual thickness $d_{v,SC}$ [m]) was thus closed for MCPA as

$$\oint \rho_B CO_2 \, d\Omega + \oint \theta C_L \, d\Omega + \oint \rho_B C_S \, d\Omega = \oint \rho_B C_T(t=0) \, d\Omega \tag{S8}$$

where $C_T(t=0)$ [μmol C/kg] is the total initial MCPA concentration.

Initial and boundary conditions were assigned as described in the main text.

2. Scale transition theory

The second order accurate approximation of the reaction rate for Monod-type kinetics is given by Eq. 7 in the main text (Chakrawal et al., 2020) as

$$\overbrace{\overline{R}(C_L, B)}^{\text{Average Reaction Rate}} = \underbrace{R(\overline{C_L}, \overline{B})}_{\text{Mean Field Approximation (MFA)}} + \overbrace{\frac{1}{2}\frac{\partial^2 R}{\partial C_L^2} var(C_L)}^{\text{Variance Term (VAR)}} + \underbrace{\frac{\partial^2 R}{\partial C_L \partial B} cov(C_L, B)}_{\text{Covariance Term (COV)}} + \sum HOT \,, \tag{S9}$$

where the partial derivatives are evaluated at the mean values of the variables ($\overline{C_L}$ and $\overline{B}$) and $\sum$HOT represents the cumulative contribution from higher order terms. Note that the additional variance term $\frac{1}{2}\frac{\partial^2 R}{\partial B^2} \mathrm{var}(B) = 0$ for Monod-type kinetics because the rate is linear with respect to B (Chakrawal et al., 2020). The reaction rate $R(C_L, B)$ in this case is given by Eq. 3 in the main text as $R(C_L, B) = \mu_{max} \frac{C_L}{K_M + C_L} B \rho_B$. Consequently, the mean field approximation (MFA), the first right hand side term in Eq. S9 (Eq. 7 in the main text) is given as

$$R(\overline{C_L},\bar{B}) = \mu_{max}\frac{\overline{C_L}}{K_M+\overline{C_L}}\bar{B}\rho_B \tag{S10}$$

which is identical with the formulation in Chakrawal et al. (2020) except for ρ_B needed here as a unit conversion factor, which carries over to the partial derivatives $\frac{\partial^2 R}{\partial C_L^2}$ [m³/μmol/s]

$$\frac{\partial^2 R}{\partial C_L^2} = -\frac{2\mu_{max}K_M\bar{B}\rho_B}{(K_M+\overline{C_L})^3} \tag{S11}$$

and $\frac{\partial^2 R}{\partial C_L \partial B}$ [kg/μmol/s]

$$\frac{\partial^2 R}{\partial C_L \partial B} = \frac{\mu_{max}K_M\rho_B}{(K_M+\overline{C_L})^2}, \tag{S12}$$

where $\bar{B}$ and $\overline{C_L}$ are the spatial averages $\left(\bar{\chi}(t) = \int\int \chi(x,y,t)\mathrm{d}x\mathrm{d}y \cdot \left(\int\int \mathrm{d}x\mathrm{d}y\right)^{-1}\right)$ of degrader and solution phase pesticide concentrations, respectively. Eq. S12 was used to assess the contribution of the covariance term to the difference between homogeneous and heterogeneous scenarios. As explained in the main text, the deviation from the mean field approximation (MFA); i.e., $\bar{R} - \mathrm{MFA}$, was compared between homogeneous and heterogeneous scenarios.

3. Measurements of soil water retention curves, hydraulic conductivities and bulk densities

We sampled undisturbed 100 cm³ soil cores from three soil layers (0-30, 30-60 and 60-90 cm; 20 replicates) by pushing stainless steel cylinders (5.6 cm diameter and 4 cm height) horizontally into the soil. The cylinders were then carefully excavated to avoid any loss of soil material on top and bottom surfaces. The soil cores were used to estimate soil water retention curves (5 replicates per depth) and saturated hydraulic conductivity (10 replicates) following standard procedures (DIN 19683-9; DIN EN ISO 11274). Bulk density was estimated by weighting oven-dried (105°C) soil cores (5 replicates) following the determination of soil water retention curves.

4. Estimation of soil hydraulic parameters

Measured soil water retention curves (SWRC) relate the pressure p [Pa] (commonly given as pF value, $pF = -\log_{10}\left(\frac{p}{100\ \mathrm{Pa}}\right)$) to the respective water content. SWRC's were obtained for three depths in the reference soil and were individually fitted using the Mualem-van Genuchten model given in Eq. S3 reformulated for θ

$$\theta = \theta_r + \frac{\theta_s - \theta_r}{(1+(\alpha_{VG}h)^{n_{VG}})^m}, \tag{S13}$$

where the pressure head h was computed from p with the fluid density $\rho_F = 1$ [kg/m³] and the gravitational constant $g = 9.81$ [m/s²] as $h = \frac{p}{\rho_F g}$. The saturated water content θ_s was directly obtained from the measurements and the hydraulic soil parameters θ_r [1], α_{VG} [1/m] and n_{VG} [1] were fitted with a hybrid global-local optimization algorithm with MATLAB®. First, MATLAB®'s `particleswarm` algorithm was used for global optimization and the found solution was then used with MATLAB®'s local `fmincon` opimization algorithm. `particleswarm` was run with a function

tolerance for termination of 1e-9 and otherwise default settings. `fmincon` was adapted to use the medium-scale `sqp` algorithm with optimality tolerance set to 1e-10 and 10,000 maximum function evaluations. Parameter bounds were 0-10^5, 1-10 and 0-θ_s for α_{VG}, n_{VG} and θ_r, respectively. All measurement points, including replicates, were fitted simultaneously and the sum of squared residuals was minimized. Best fits to the experimentally measured SWRC are shown in Figure S1. Best fitting parameters are given in Table 1 in the main text.

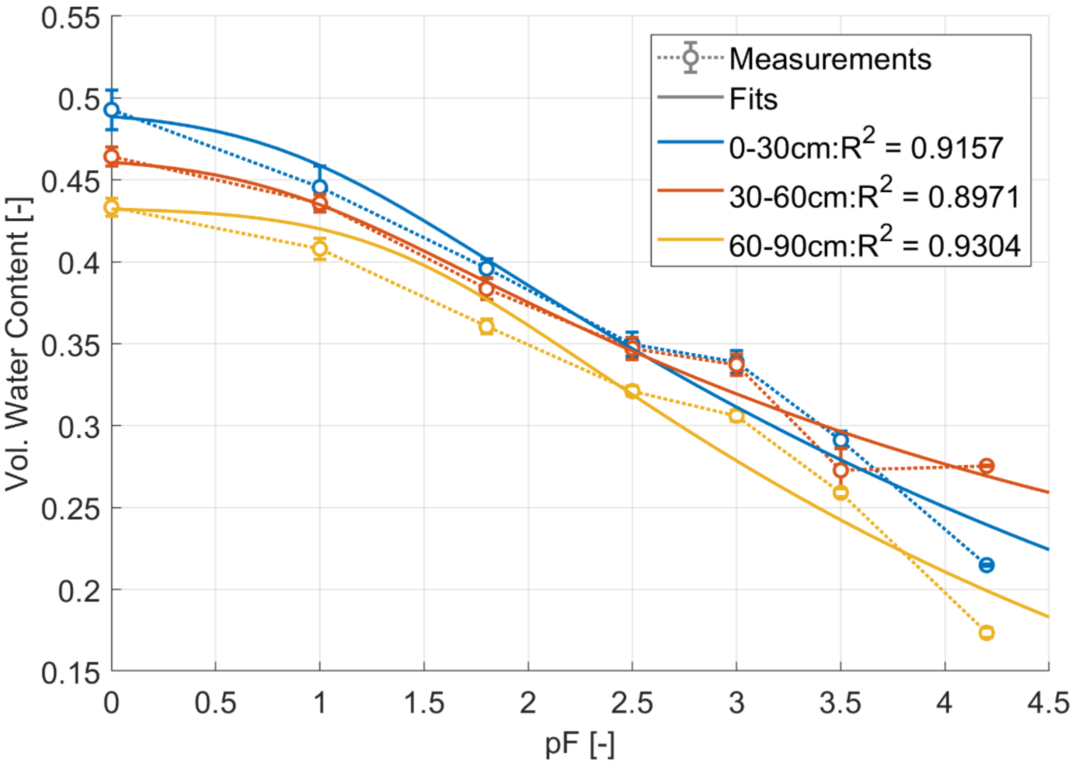

Figure S1 Fitted SWRC against measured data in depths of 0-30 cm, 30-60 cm, and 60-90 cm. Open circles mark means of replicates and error bars indicate the standard error of the mean (n=5 for pF 0 – 3.5 and n=4 for pF 4.2) Solid lines show best fits of the Mualem-van Genuchten retention model to the data.

5. Fitting of the MCPA degradation model

A process-based model that considers non-linear pesticide sorption and biodegradation was fitted to MCPA degradation data from recent microcosm experiments using the reference soil (Wirsching et al., 2020). The model accounted for Freundlich equilibrium sorption and Monod-type degradation kinetics by the following ordinary differential equation of the solution phase MCPA concentration:

$$\frac{dC_L}{dt} = -\frac{1}{RF}\mu_{max}\frac{C_L}{K_M + C_L}B_{TS}\frac{\rho_{B,E}}{\theta_E}. \tag{S14}$$

Diverging from the simulations with transient water content presented in the main text, experiments were run at a constant volumetric water content $\theta_E = 0.25$ and soil bulk density $\rho_{B,E} = 1200$ kg/m^3 (Wirsching et al., 2020). Thus, equilibrium sorption was considered by the retardation factor

$$RF = 1 + K_F n_F (C_L)^{n_F - 1}\frac{\rho_{B,E}}{\theta_E}. \tag{S15}$$

Wirsching et al. (2020) measured MCPA degradation by the indigenous microbial population in soil samples of the reference soil (an arable Luvisol; SM3, CAMPOS; for further details on soil properties and soil sampling see Wirsching et al. (2020)). In brief, *tfdA* gene abundance was measured as a proxy of the microbial degradation potential and degradation was assessed at different levels of initial MCPA concentration (0, 0.03, 0.05, 0.1, 0.5, 1, 5, 20 mg/kg). Since *tfdA* genes only slightly increased in response to highest amendment of the experiment, we used the average *tfdA* abundance ($1.11 \cdot 10^8$ genes/kg) in the control treatment as a proxy for the MCPA degradation potential in the topsoil (B_{TS} [μmol C / kg] $= 1.11 \cdot 10^8$ genes/kg $\cdot f_{m/g}$). Timeseries of total residual pesticide concentration in the two batch experiments with the highest initial MCPA concentrations (5 and 20 mg/kg) were fitted with the same set of parameters. The differential equation in Eq. S14 was integrated with MATLAB®'s `ode45` with relative and absolute tolerance set to 10^{-10} and 10^{-9}, respectively. As before for fitting SWRCs, all individual measurement points, including replicates, were fitted simultaneously. To better represent degradation dynamics at low concentration levels, all values were $\log_{10}$-transformed before computing sums of squared residuals. μ_{max} and K_M where constrained to values between $10^{-6} - 10^3$ and $10^{-3} - 10^9$, respectively.

Fitting $\log_{10}$-transformed data was motivated by the better representation of degradation dynamics at low residual concentrations but caused initial degradation to be overestimated (compare supplementary Figure S2: original data (grey circles) vs. fit (red line)). This mismatch with experimental observations, however, does not impede inter-scenario comparisons.

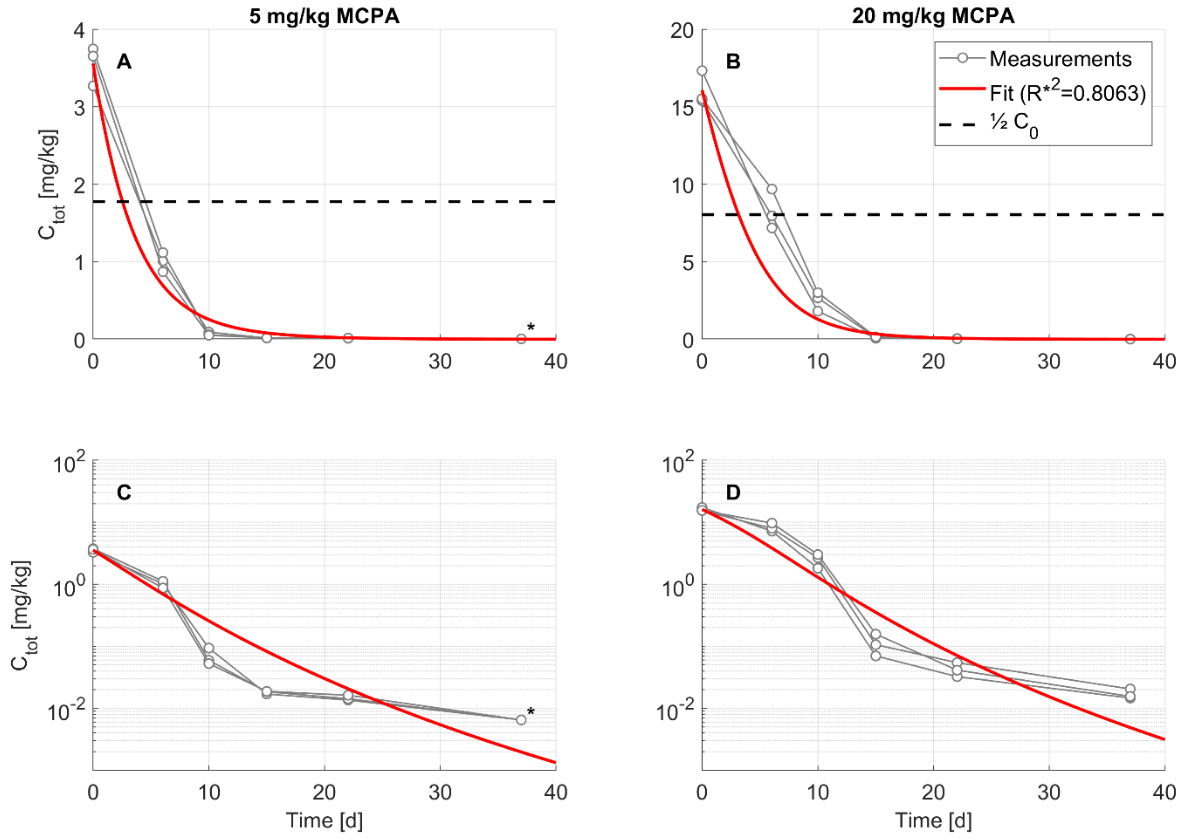

Figure S2 Fits of degradation kinetics to experimental data for experiments with initial MCPA concentrations of 5 mg/kg (A,C) and 20 mg/kg (B,D) (Wirsching et al., 2020). Data was fitted to $\log_{10}$-transformed residual concentration data (C,D).The black asterix in (A) and (C) mark data points below the limit of quantification (LOQ = 13 µg/kg; Wirsching et al., 2020) which where set to half of the LOQ. Dashed lines in (A, B) are isolines indicating $C = \frac{1}{2} C_0$.

6. Distribution properties

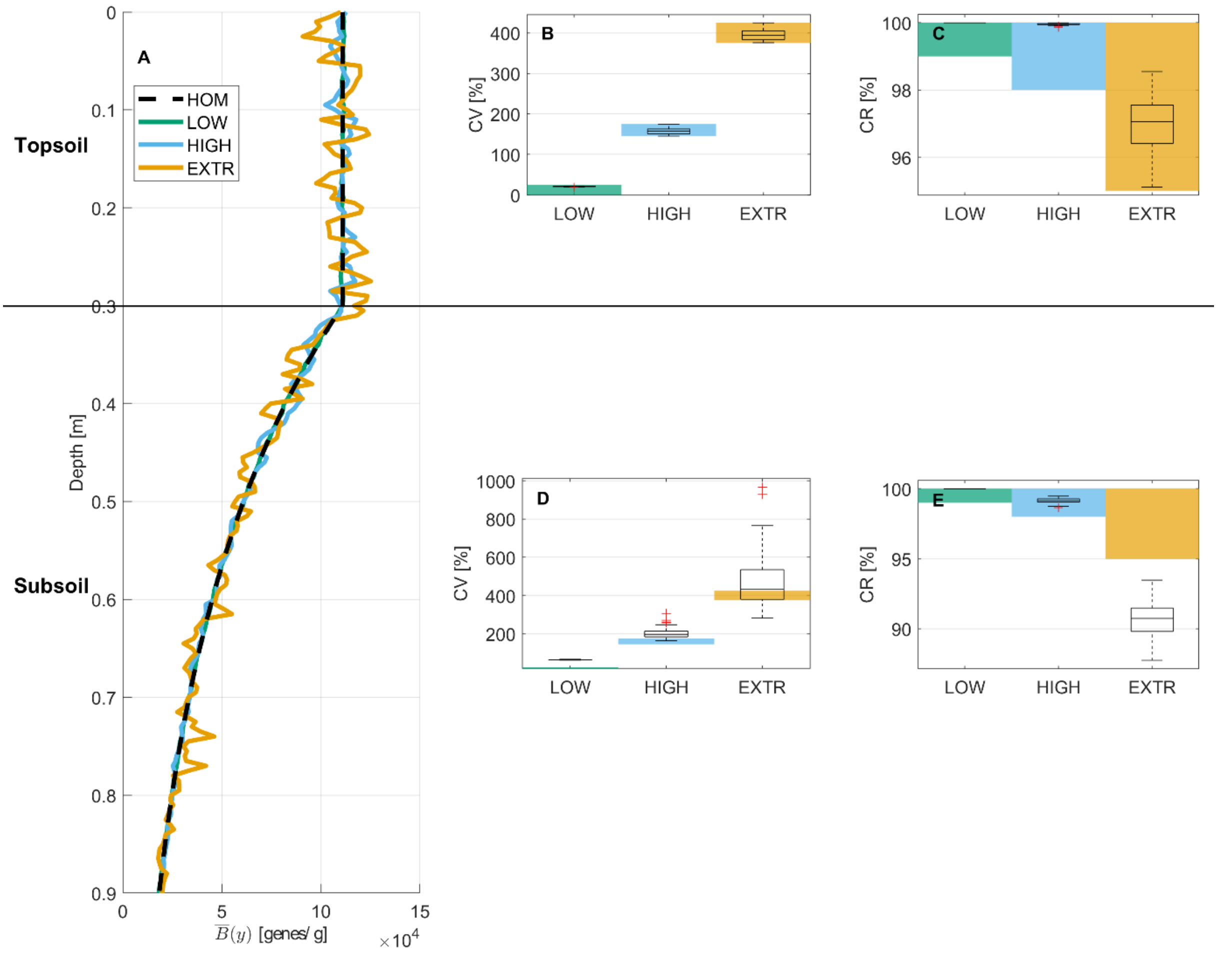

Figure S3 Distribution statistics of created distributions. (A) Ensemble mean of average vertical profiles of degrader abundances ($\hat{B}(y)$) of all stochastic distributions of each heterogeneity scenario. HOM follows the function given by Eq. 4 in the main text. (B, D) Coefficients of variation (CV [%]) in the top- and subsoil, respectively. (C, E) Colonization ratio of soil patches (CR [%], samples were considered uncolonized if $B < 100$ genes/g) in the top- and subsoil, respectively. Shaded areas mark the established limits at which individual simulations were accepted in the respective scenarios (limits were applied to the topsoil only).

7. Distribution examples

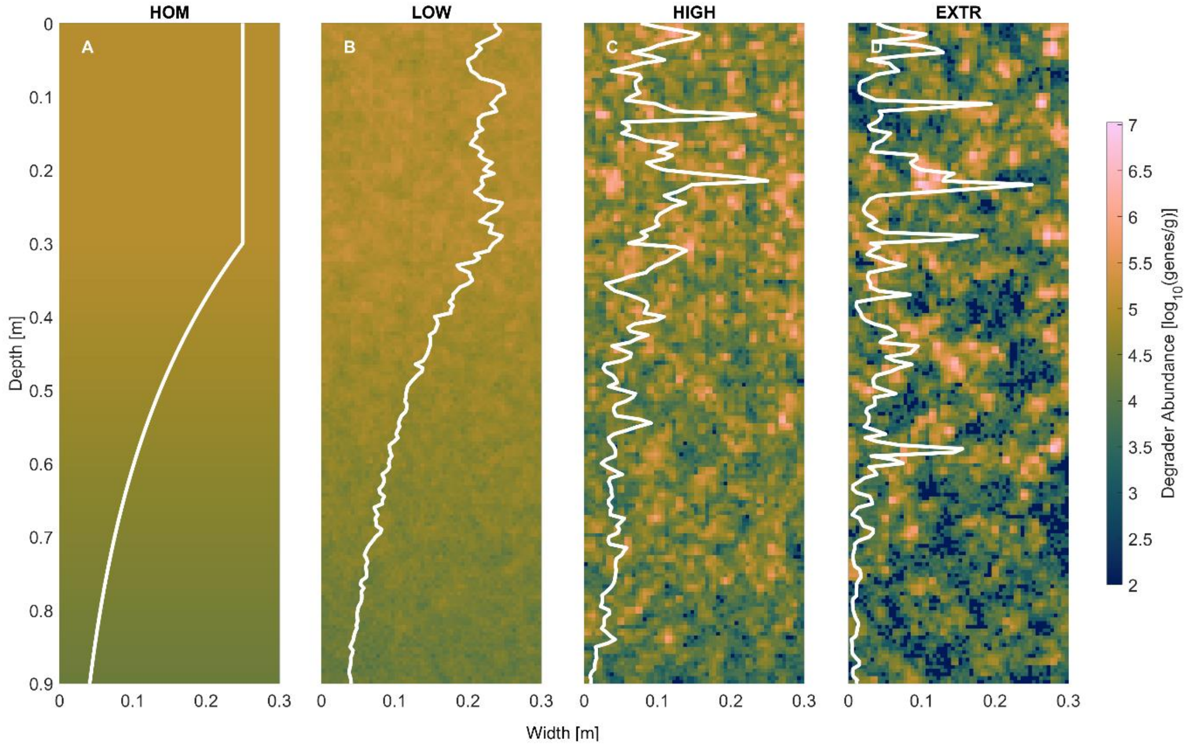

Figure S4 Examples of spatial distributions of degrader abundances (log$_{10}$-transformed values) of the four heterogeneity scenarios. White lines indicate average degrader abundances as a function of depth ($\hat{B}_i(y)$) (scaled as $\hat{B}_i(y)/max(\hat{B}_i(y)) \cdot 0.25$, where i is HOM, LOW, HIGH, or EXTR). $\hat{B}_{HOM}(y)$ follows the function given by Eq. 4 in the main text.

8. Model exploration for different dispersivity values and ratios

Soil dispersivity values (Eq. S5) are highly uncertain (Vanderborght & Vereecken, 2007). We thus tested the sensitivity of half-lives (DT50, time when 50% of the initially applied pesticide dissipated from the entire simulation domain) to changes in λ_L from 0.01 to 0.1 m (Vanderborght & Vereecken, 2007) at $\frac{\lambda_L}{\lambda_T}$ ratios of 3 and 10 (default values were: $\lambda_L = 0.03$ m and $\lambda_L/\lambda_T = 3$, see Table 1 in the main text). DT50 values in HOM and LOW hardly changed over the explored dispersivity range (supplementary Figure S5). Due to the increased mixing of substrate in EXTR (and to a lesser extend in HIGH), DT50 decreased with increasing dispersivities (both, λ_L and λ_T). Intra-scenario variation of DT50 with the default dispersivity settings, however, largely exceeded the variation observed for the range of tested dispersivities in a single distribution realization (supplementary Figure S5 B, D).

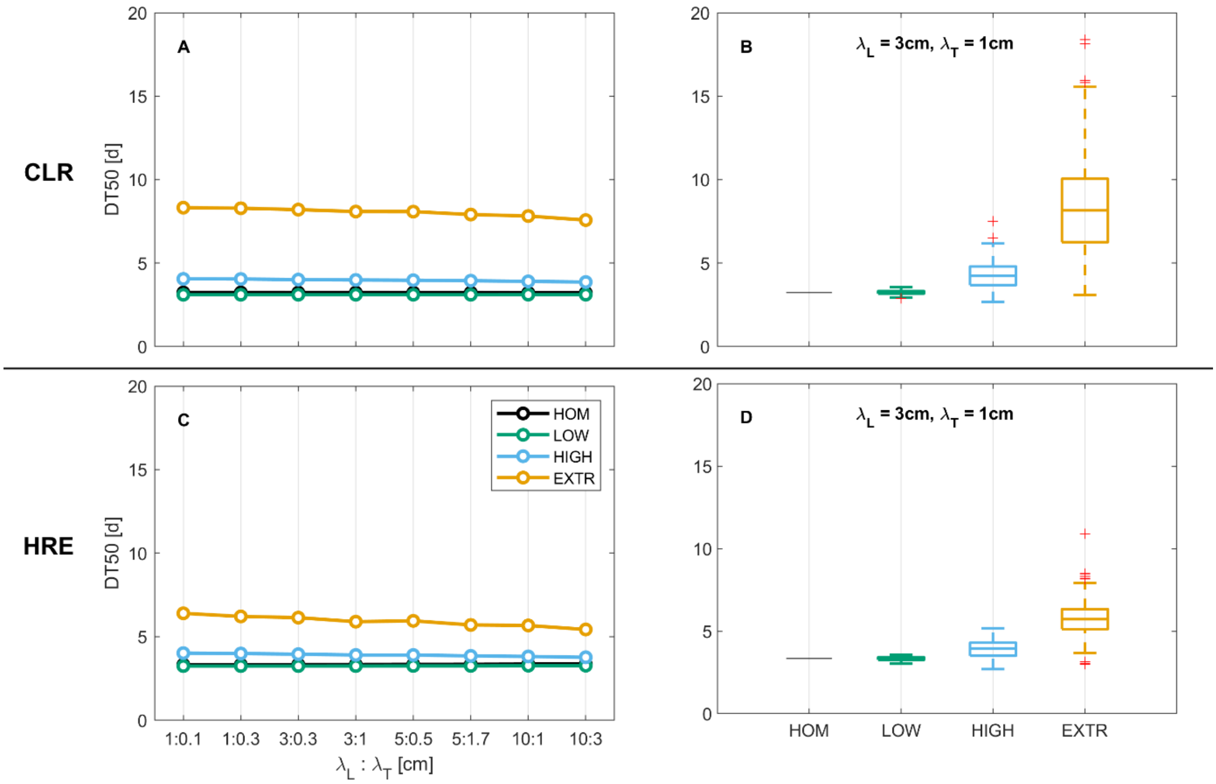

Figure S5 Sensitivity of DT50 values of all scenarios to variation in longitudinal and transversal dispersivities (λ_L [cm] and λ_T [cm] respectively) and their ratio (A, C) in continuous light rain (CLR; A, B) and heavy rain events (HRE; C, D) scenarios. Per scenario, a single realization of a degrader distribution was chosen. The variation in DT50 between the 100 stochastic simulations in each heterogeneity scenario with the default dispersivities is depicted as boxplots (B, D).

9. Leachate concentration and cumulatively leached MCPA load

Figure S6 shows the same data as Figure 2 in the main text but with logarithmic y-axes.

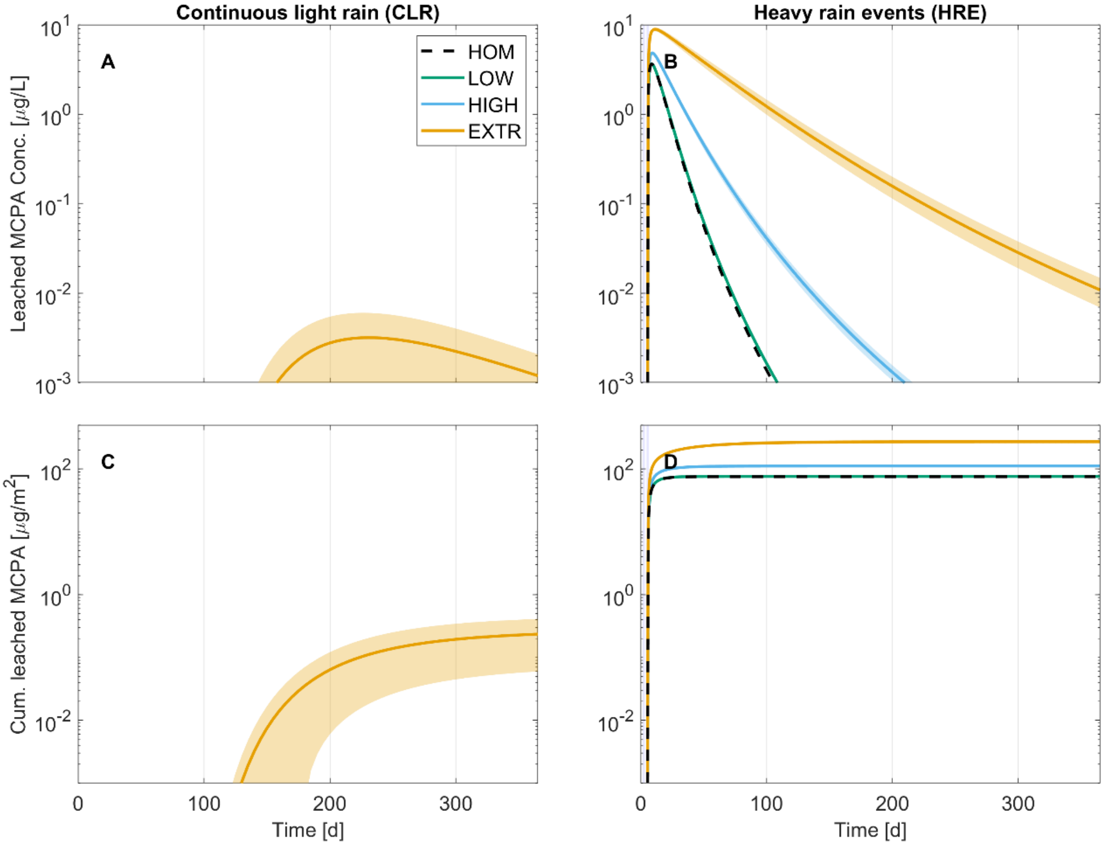

Figure S6 Time series of averaged MCPA leachate concentration (A, B) and cumulatively leached MCPA load (C, D) from the topsoil at 30 cm depth in continuous light rain (CLR; A, C) and heavy rain events (HRE; B, D) scenarios. Lines represent scenario means and shaded areas mark their 99 %-confidence intervals. Blue bars in panels B & D indicate when heavy rain events occurred. Note that y-axes are logarithmic. This figure shows the same data as Figure 2 in the main text.

10. Temporal evolution of scale transition terms

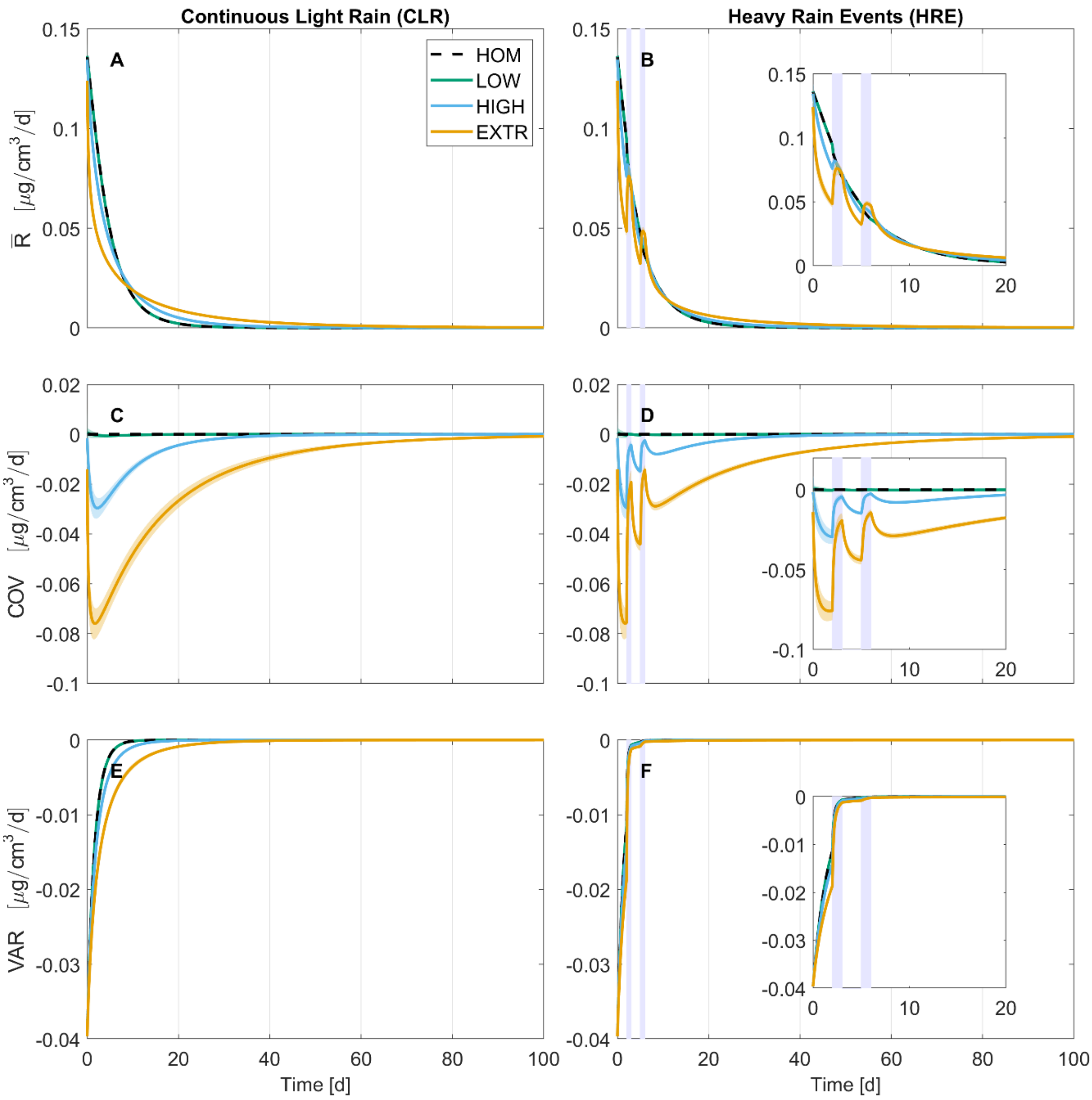

Figure S7 Temporal evolution of $\overline{R}$ (A, B), COV (C, D) and VAR (E, F) in continuous light rain (CLR; A, C, E) and heavy rain events (HRE; B, D, F) scenarios. Lines represent scenario means and shaded areas mark their 99%-confidence intervals. Inserts in panels (B, D, F) zoom in on dynamics at days 0-20. Blue bars indicate when heavy rain events occurred.

11. Dimensionless scale transition approach

Wilson and Gerber (2021) recently suggested a dimensionless reformulation of the scale transition approach by Chakrawal et al. (2020). In brevity, Wilson and Gerber (2021, Eq. 17, p. 5672) suggest to express Eq. S9 (Eq. 7 in the main text) truncated to second order accuracy (i.e., neglecting $\sum HOT$) in a dimensionless form which, applied to the Monod-type kinetics we assumed corresponds to

$$\overbrace{\frac{\bar{R}(C_L, B) - MFA}{MFA}}^{Scale\ Transition\ Correction} \approx \left(CV(C_L)\right)^2 \frac{1}{1+\lambda}\left(\rho_{C_L,B}\lambda_2 - \frac{\lambda}{1+\lambda}\right) , \tag{S16}$$

where $CV(C_L) = \frac{var(C_L)}{\overline{C_L}^2}$ [1] and $CV(B) = \frac{var(B)}{\bar{B}^2}$ [1] are the coefficients of variation of the dissolved phase substrate concentration C_L and microbial degrader biomass B, respectively. $\rho_{C_L,B}$ [1] is the correlation coefficient between C_L and B, and the dimensionless ratios λ and λ_2 are defined as $\lambda = \frac{\overline{C_L}}{K_M}$ [1] and $\lambda_2 = \frac{CV(B)}{CV(C_L)}$ [1]. Expressing Eq. S16 in terms of $CV(B)$ yields

$$\overbrace{\frac{\bar{R}(C_L, B) - MFA}{MFA}}^{Scale\ Transition\ Correction} \approx \left(CV(B)\right)^2 \frac{1}{1+\lambda}\left(\frac{\rho_{C_L,B}}{\lambda_2} - \frac{\lambda}{(1+\lambda){\lambda_2}^2}\right) . \tag{S17}$$

These formulations allow for analytical examination of how $CV(B)$, respectively $CV(C_L)$ impact the systems deviation from the mean field approximation (i.e., the scale transition correction) for specific combinations of $\lambda, \rho_{C_L,B}$, and λ_2 values. In our simulations all these variables (with the exception of $CV(B)$) vary through time. How $CV(B)$ and $CV(C_L)$ influence the scale transition correction thus changes throughout the course of the simulation, and between heterogeneity scenarios. The analysis presented in the main text, based on the covariance term, accounts for these interactions as the covariance between degrader and substrate distribution can be expressed as

$$cov(C_L, B) = \rho_{C_L,B} CV(B) CV(C_L) \bar{B}\overline{C_L} , \tag{S18}$$

similar to Eq. 9 in Wilson and Gerber (2021, p. 5671). Despite the multiple sources of variability, the dimensionless depiction of simulation outcomes illustrates that the relative deviation from the MFA (the scale transition correction) does not diminish as substrate is used up over time, but that a stable deviation is reached in the order HOM<LOW<HIGH<EXTR (Figure S8).

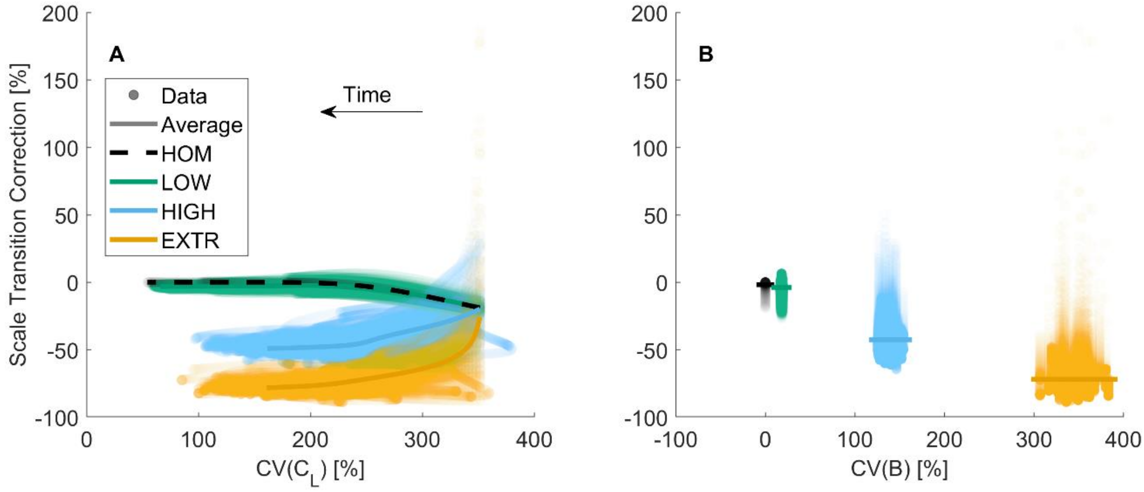

Figure S8 Dimensionless scale transition correction as a function of CV(C_L) (A) and CV(B) (B) for all continuous light rain (CLR) scenarios. Circles represent all data points (100 simulations x 355 timepoints per heterogeneity scenario) and thick lines the scenario mean (per time point in A and over all data in B). CV(C_L) declines with time, thus in panel A time goes in the opposite x-axis direction as indicated by the arrow. Note that the CV(B) values shown in panel B were slightly shifted to lower values compared to the generated distributions (Fig. S3 B) due to their spatial interpolation to the finite element mesh in COMSOL®.

12. References

Chakrawal, A.; Herrmann, A. M.; Koestel, J.; Jarsjö, J.; Nunan, N.; Kätterer, T.; Manzoni, S. Dynamic upscaling of decomposition kinetics for carbon cycling models. *Geoscientific Model Development* **2020**, *13*, 1399–1429, doi.org/10.5194/gmd-13-1399-2020.

DIN 19683-9 (2012). Bodenbeschaffenheit – Physikalische Laboruntersuchungen – Teil 9: Bestimmung der Wasserdurchlässigkeit in wassergesättigten Stechzylinderbodenproben. *Beuth Verlag GmbH,* DIN 19683-9:2012-07.

DIN EN ISO 11274 (2020), Bodenbeschaffenheit – Bestimmung des Wasserrückhaltevermögens – Laborverfahren (ISO_11274:2019); Deutsche Fassung EN ISO 11274:2019. *Beuth Verlag GmbH,* DIN EN ISO 11274: 2020-04.

Millington, R.; Quirk, J. Permeability of porous solids. *Transactions of the Faraday Society* **1961**, *57*, 1200–1207, doi.org/10.1039/TF9615701200.

Mualem, Y. A new model for predicting the hydraulic conductivity of unsaturated porous media. *Water resources research* **1976**, *12*, 513–522, doi.org/10.1029/WR012i003p00513.

Vanderborght, J.; Vereecken, H. Review of dispersivities for transport modeling in soils. *Vadose Zone Journal* **2007**, *6*, 29–52, doi.org/10.2136/vzj2006.0096.

Wilson, C. H.; Gerber, S. Theoretical insights from upscaling Michaelis–Menten microbial dynamics in biogeochemical models: a dimensionless approach. *Biogeosciences* **2021**, *18*, 5669–5679, doi.org/10.5194/bg-18-5669-2021.

Wirsching, J.; Pagel, H.; Ditterich, F.; Uksa, M.; Werneburg, M.; Zwiener, C.; Berner, D.; Kandeler, E.; Poll, C. Biodegradation of Pesticides at the Limit: Kinetics and Microbial Substrate Use at Low Concentrations. *Frontiers in microbiology* **2020**, *11*, 2107, doi.org/10.3389/fmicb.2020.02107.
