# Supplementary material for: Spatial Control of Microbial Pesticide Degradation in Soil: A Model-Based Scenario Analysis
Source: Environ Sci Technol. 2022 Sep 27;56(20):14427–38. doi: 10.1021/acs.est.2c03397 (PMC9583605; doi:10.1021/acs.est.2c03397)
Supplement: Supplementary file 2 — es2c03397_si_002.pptx [file es2c03397_si_002.pptx]

## Slide 1
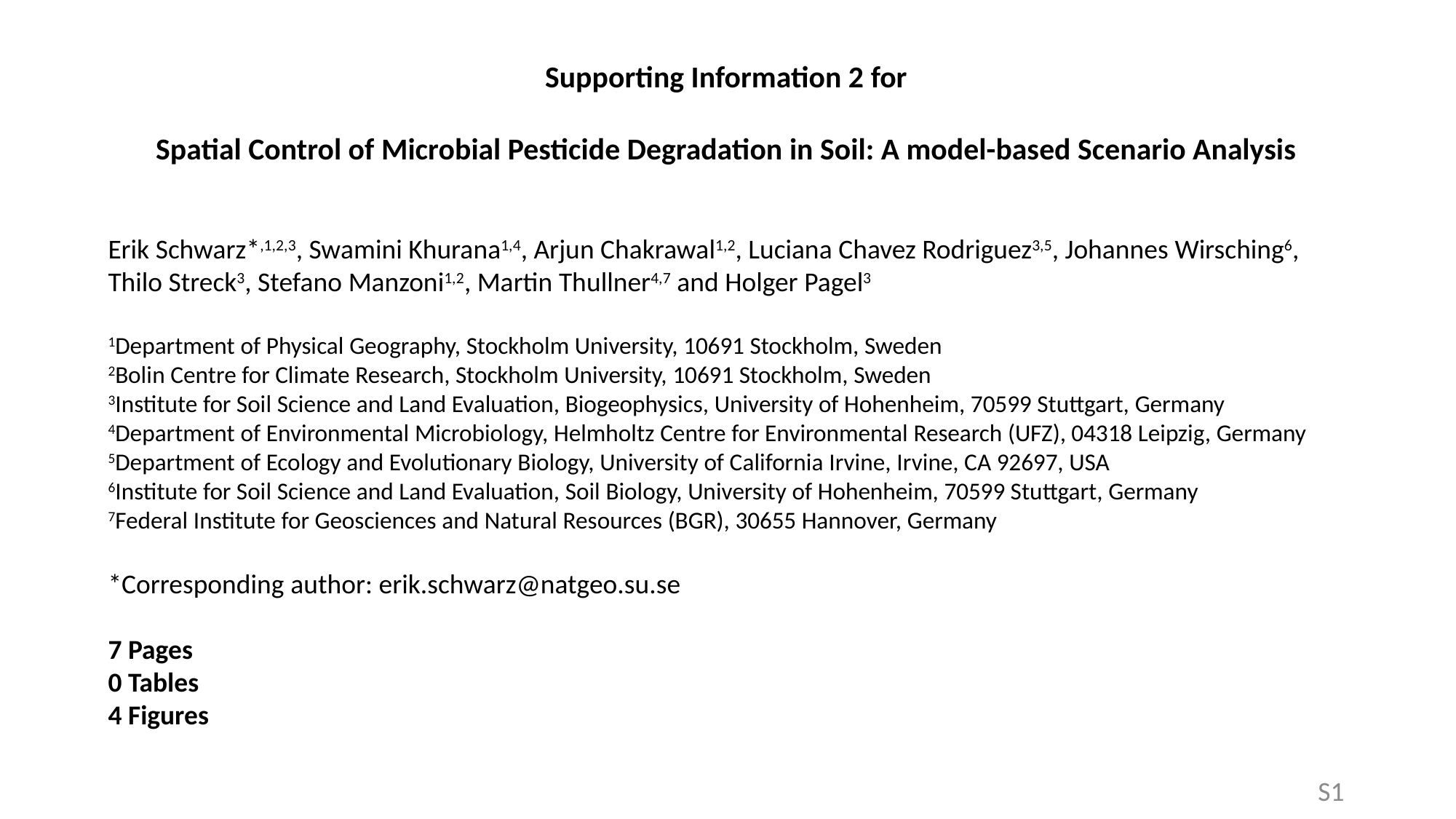

Supporting Information 2 for
Spatial Control of Microbial Pesticide Degradation in Soil: A model-based Scenario Analysis
Erik Schwarz*,1,2,3, Swamini Khurana1,4, Arjun Chakrawal1,2, Luciana Chavez Rodriguez3,5, Johannes Wirsching6, Thilo Streck3, Stefano Manzoni1,2, Martin Thullner4,7 and Holger Pagel3
1Department of Physical Geography, Stockholm University, 10691 Stockholm, Sweden
2Bolin Centre for Climate Research, Stockholm University, 10691 Stockholm, Sweden
3Institute for Soil Science and Land Evaluation, Biogeophysics, University of Hohenheim, 70599 Stuttgart, Germany
4Department of Environmental Microbiology, Helmholtz Centre for Environmental Research (UFZ), 04318 Leipzig, Germany
5Department of Ecology and Evolutionary Biology, University of California Irvine, Irvine, CA 92697, USA
6Institute for Soil Science and Land Evaluation, Soil Biology, University of Hohenheim, 70599 Stuttgart, Germany
7Federal Institute for Geosciences and Natural Resources (BGR), 30655 Hannover, Germany
*Corresponding author: erik.schwarz@natgeo.su.se
7 Pages
0 Tables
4 Figures
S1

## Slide 2
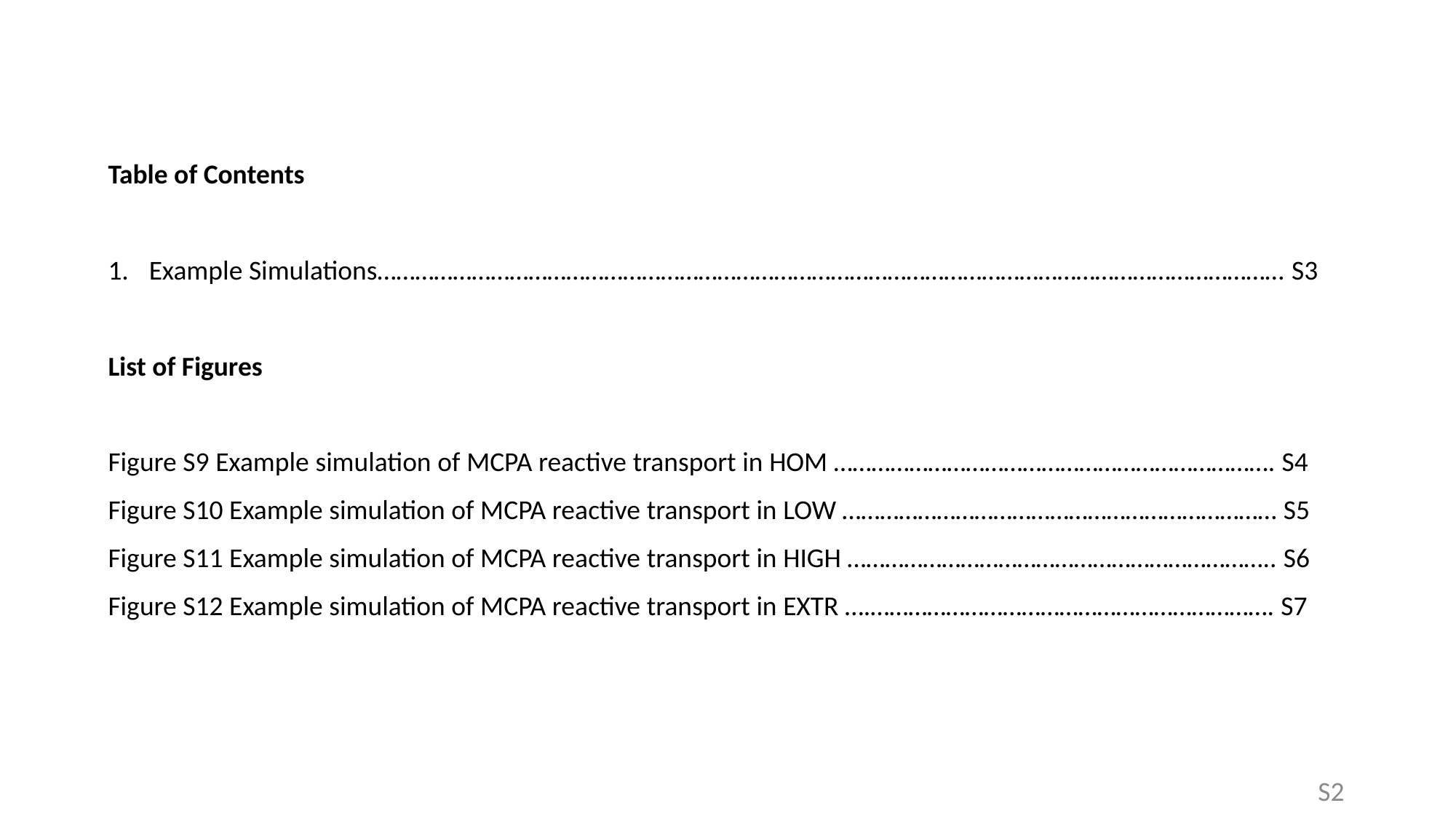

Table of Contents
Example Simulations……………………………………………………………………………………………………………………………… S3
List of Figures
Figure S9 Example simulation of MCPA reactive transport in HOM ……………………………………………………………. S4
Figure S10 Example simulation of MCPA reactive transport in LOW …………………………………………………………… S5
Figure S11 Example simulation of MCPA reactive transport in HIGH ………………………………………………………….. S6
Figure S12 Example simulation of MCPA reactive transport in EXTR ….………………………………………………………. S7
S2

## Slide 3
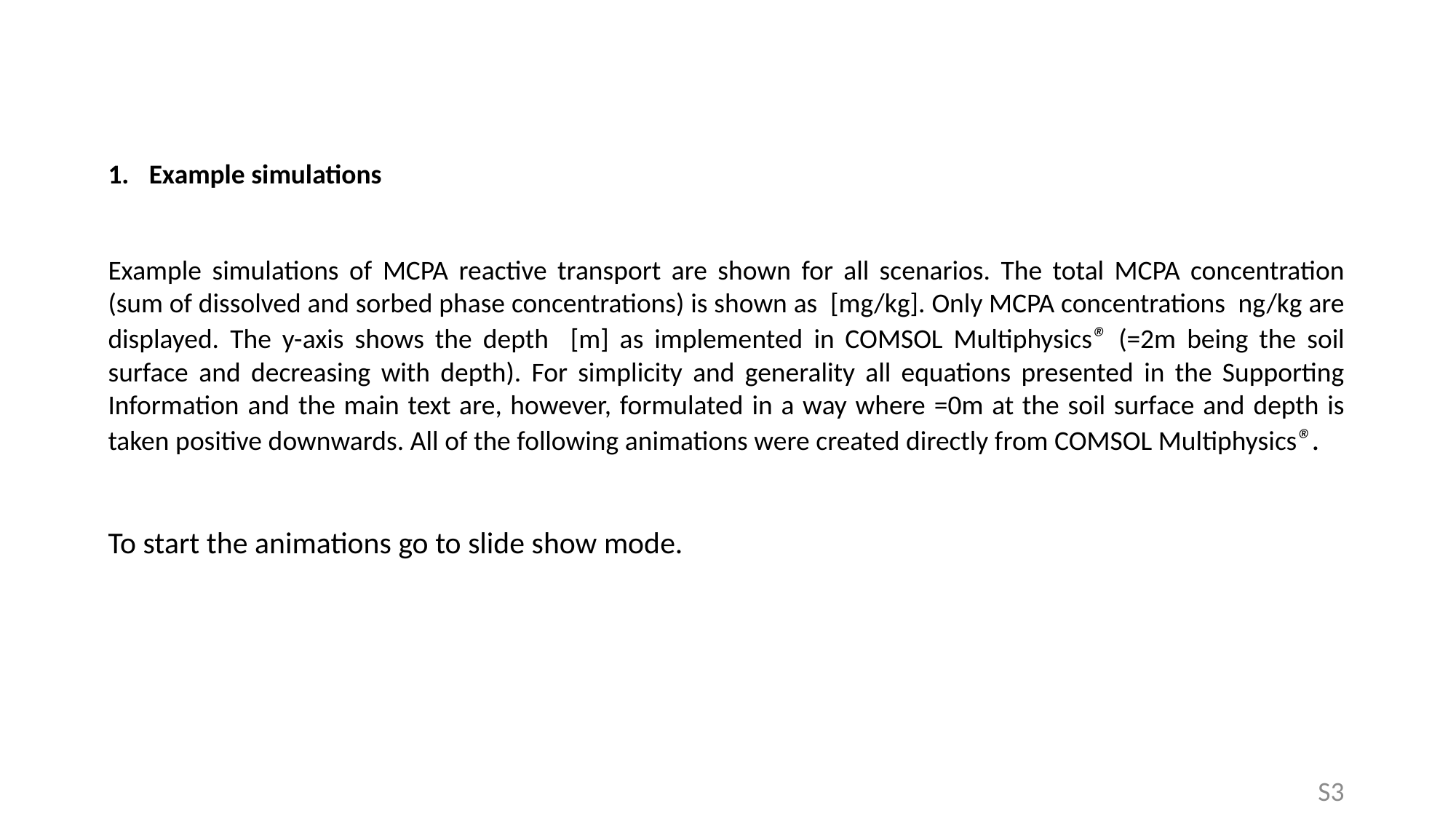

S3

## Slide 4
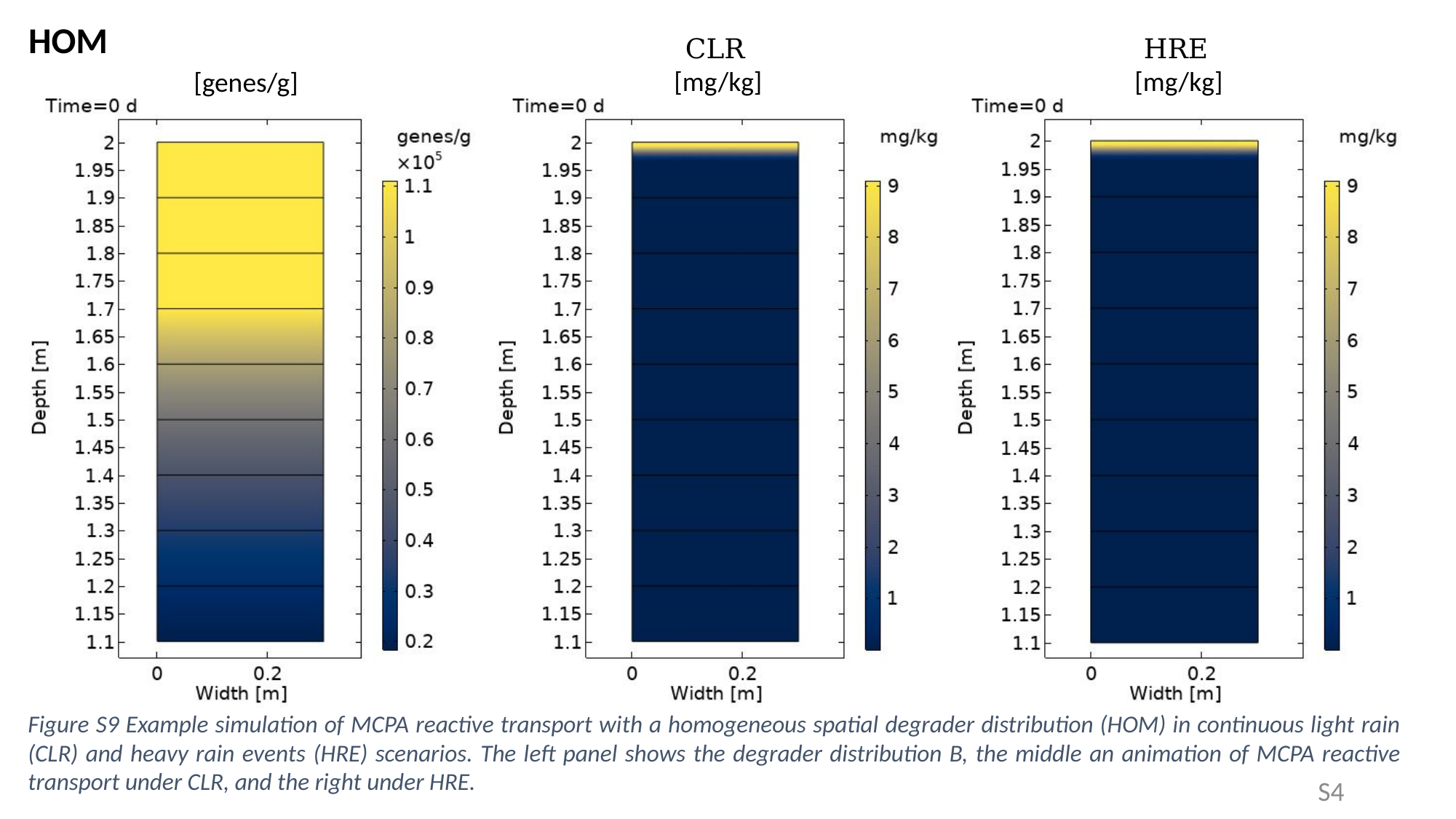

HOM
Figure S9 Example simulation of MCPA reactive transport with a homogeneous spatial degrader distribution (HOM) in continuous light rain (CLR) and heavy rain events (HRE) scenarios. The left panel shows the degrader distribution B, the middle an animation of MCPA reactive transport under CLR, and the right under HRE.
S4

## Slide 5
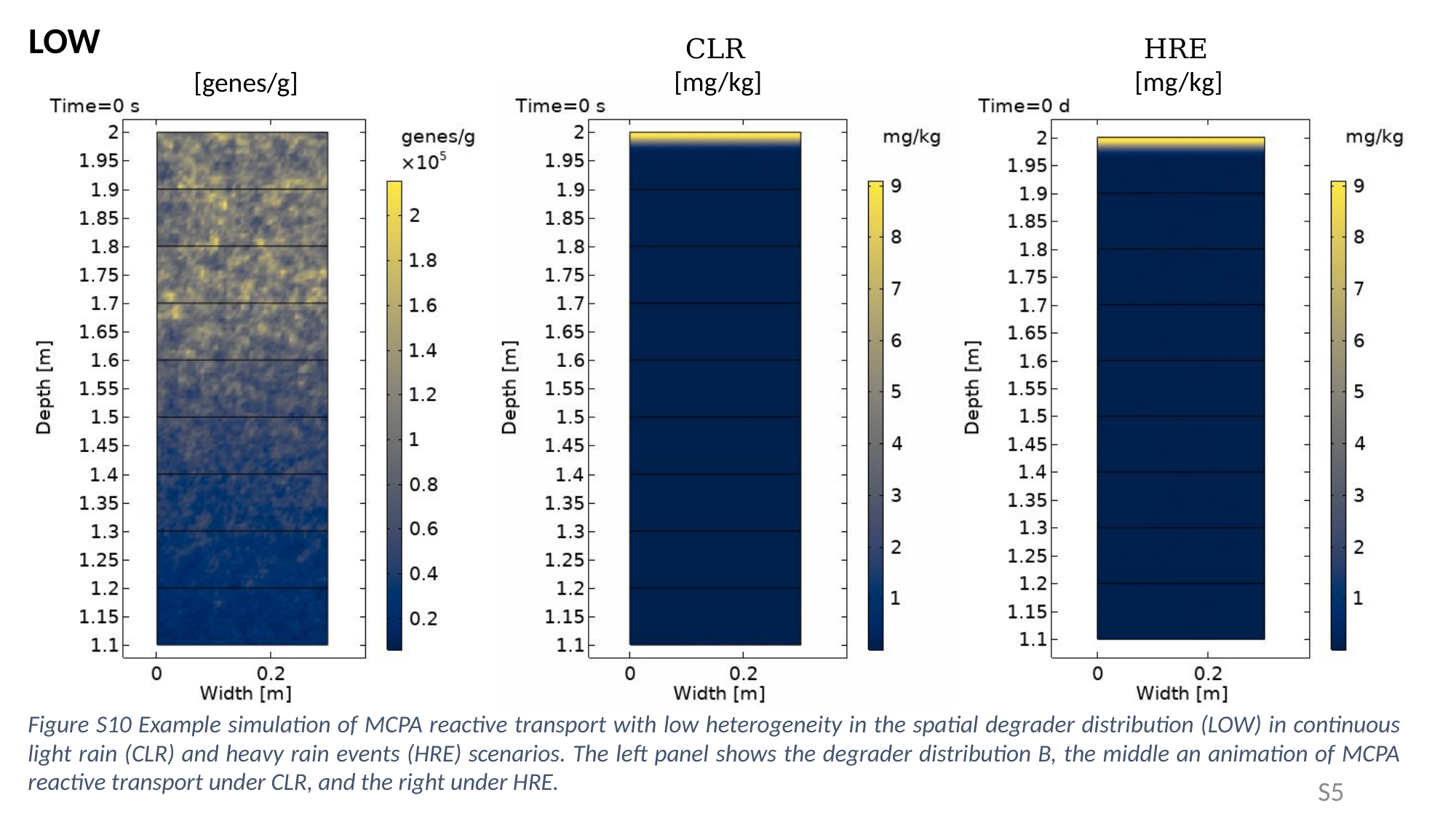

LOW
Figure S10 Example simulation of MCPA reactive transport with low heterogeneity in the spatial degrader distribution (LOW) in continuous light rain (CLR) and heavy rain events (HRE) scenarios. The left panel shows the degrader distribution B, the middle an animation of MCPA reactive transport under CLR, and the right under HRE.
S5

## Slide 6
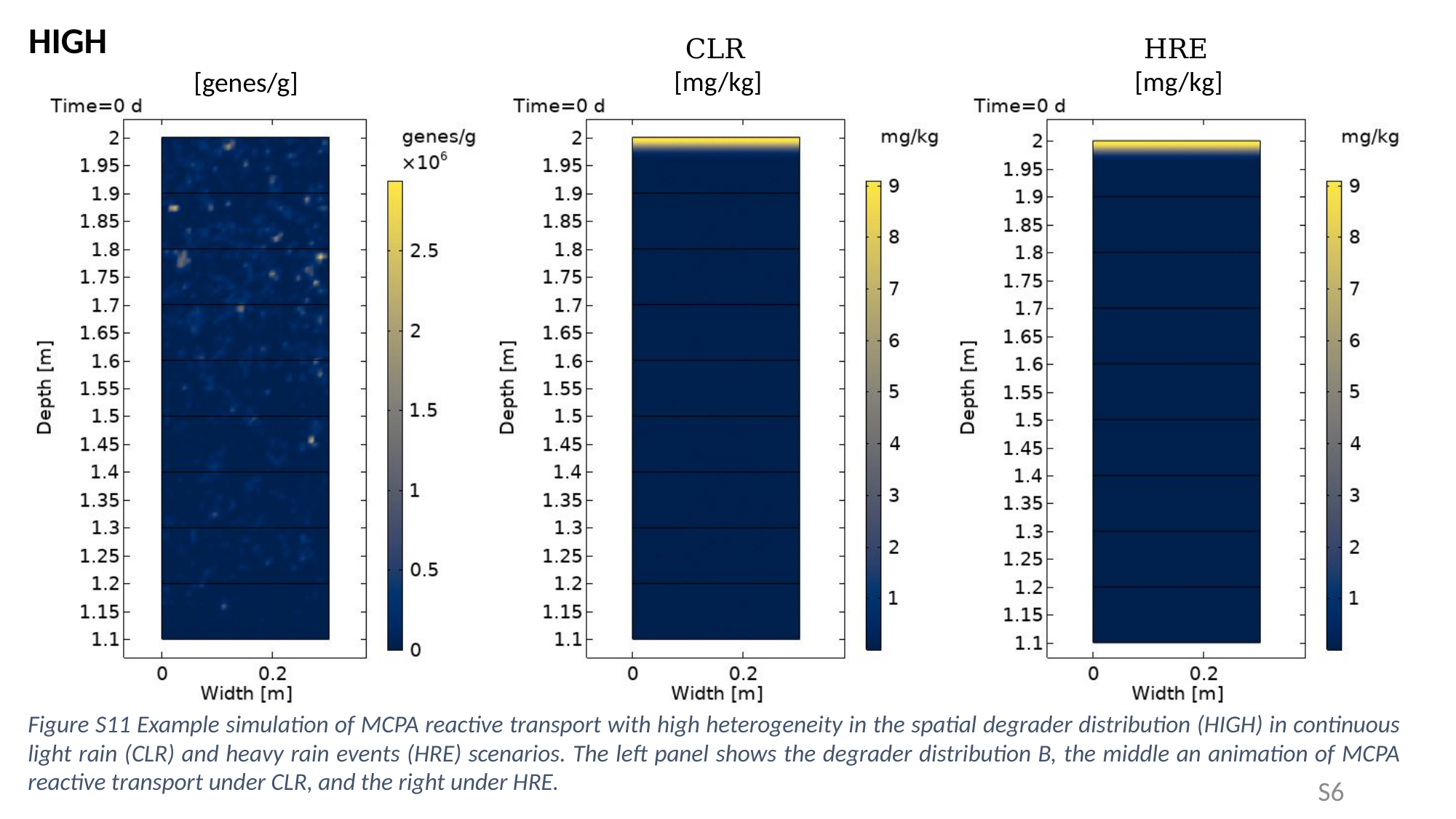

HIGH
Figure S11 Example simulation of MCPA reactive transport with high heterogeneity in the spatial degrader distribution (HIGH) in continuous light rain (CLR) and heavy rain events (HRE) scenarios. The left panel shows the degrader distribution B, the middle an animation of MCPA reactive transport under CLR, and the right under HRE.
S6

## Slide 7
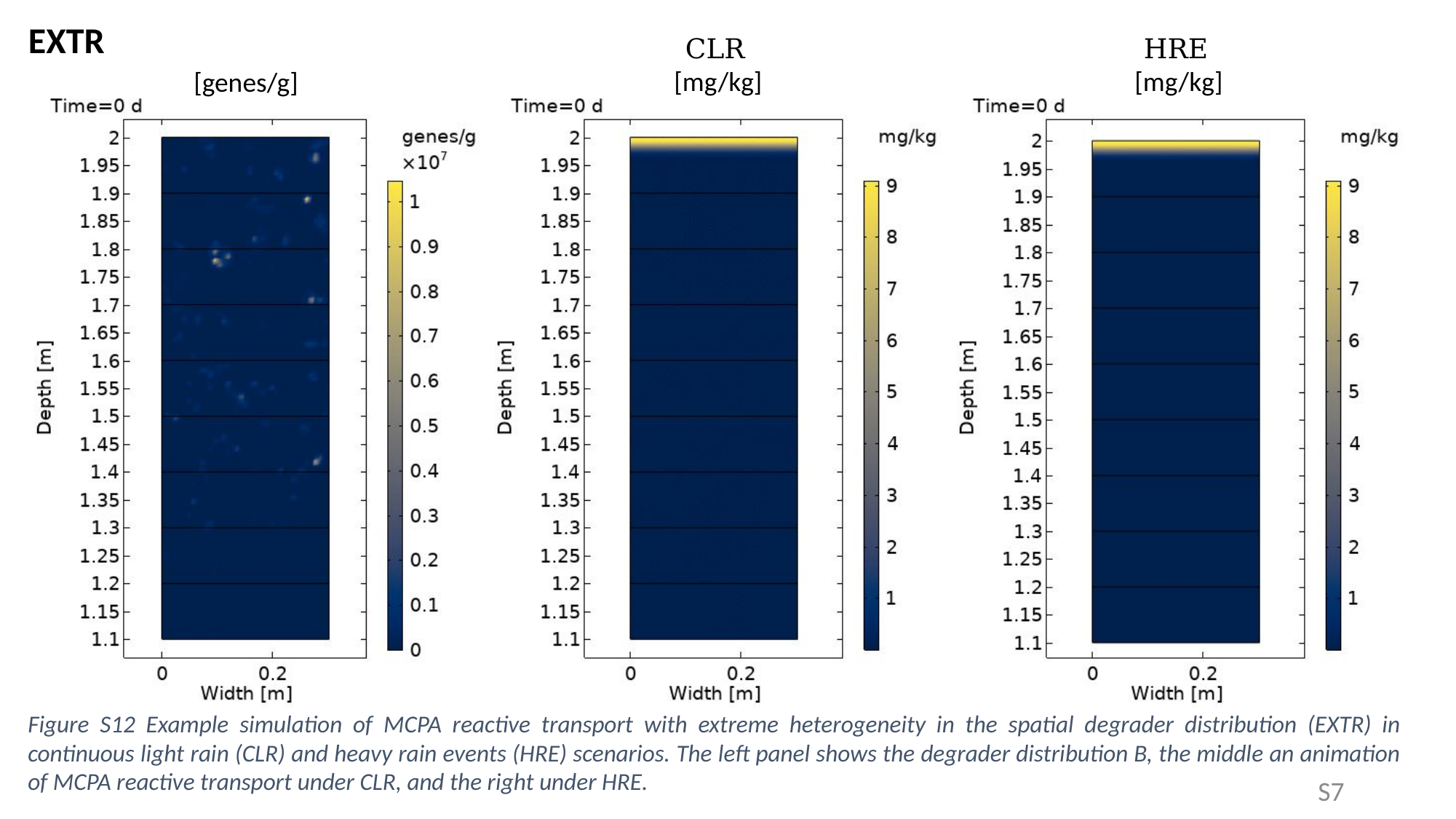

EXTR
Figure S12 Example simulation of MCPA reactive transport with extreme heterogeneity in the spatial degrader distribution (EXTR) in continuous light rain (CLR) and heavy rain events (HRE) scenarios. The left panel shows the degrader distribution B, the middle an animation of MCPA reactive transport under CLR, and the right under HRE.
S7
